# Natural–Synthetic Hybrid Nanostructures Formed Through the Interaction of Chitosan with Carboxylate-Ended PNIPAM: Structure and Curcumin Encapsulation †

**DOI:** 10.3390/nano15050350

**Published:** 2025-02-24

**Authors:** Elena-Daniela Lotos, Maria Karayianni, Ana-Lavinia Vasiliu, Marcela Mihai, Stergios Pispas

**Affiliations:** 1 Petru Poni Institute of Macromolecular Chemistry, 41A Grigore Ghica Voda Alley, 700487 Iasi, Romania; daniela.lotos@icmpp.ro (E.-D.L.); m.karayianni@icmpp.ro (M.K.); vasiliu.lavinia@icmpp.ro (A.-L.V.); 2 Theoretical and Physical Chemistry Institute, National Hellenic Research Foundation, 48 Vassileos Constantinou Ave., 116 35 Athens, Greece

**Keywords:** chitosan, poly(*N*-isopropylamide), electrostatic complexation, hybrid nanostructures, thermoresponsiveness, drug delivery, curcumin

## Abstract

Chitosan is widely used in drug delivery applications, due to its biocompatibility, bio-degradability, and low toxicity. Nevertheless, its properties can be enhanced through the physical or chemical modification of its amino and hydroxyl groups. This work explores the electrostatic complexation of two chitosan samples of differing lengths with two poly(*N*-isopropylacrylamide) (PNIPAM) homopolymers of different molecular weight carrying a chargeable carboxyl end group. This interaction enables the electrostatic binding of PNIPAM side chains onto the chitosan backbone through the amino groups, and could be considered as an alternative grafting method. Dynamic and electrophoretic light scattering techniques were employed in order to study the solution/dispersion properties of the formed complexes as a function of the PNIPAM concentration, or, equivalently, the molar/charge ratio of the two components. The obtained results revealed that their mass, size, and charge mostly depend on the length of the two individual constituents, as well as their mixing ratio. Furthermore, their response to changes in their environment, namely temperature and ionic strength, was also examined, demonstrating the effect of either the thermoresponsiveness of PNIPAM or the electrostatic charge screening, respectively. Fluorescence spectroscopy, utilizing pyrene as a probe, provided information regarding the hydrophobicity of the formed complexes, while images from scanning transmission electron and atomic force microscopies further elucidated their morphology, which was found to be closely related to that of the corresponding chitosan molecule. Finally, their potential as drug delivery vehicles was also investigated, utilizing curcumin as a model drug at various loading concentrations.

## 1. Introduction

Drug delivery technologies have advanced significantly in recent years, enabling the formulation of a wide range of pharmaceutical products that boost patient health by ensuring the precise delivery of therapeutics to their designated sites, limiting unintended distribution, and supporting patient compliance [1]. The design of suitable nanocarriers is especially crucial since they can protect drugs from premature degradation, improve their bioavailability, prolong the residence of drugs in desired anatomic sites, and thus reduce required doses and extend administration intervals [2]. Lately, polymer-based drug delivery systems, utilizing either naturally occurring or man-made synthetic polymers, have attracted considerable interest because they are cost effective and also result in promising outcomes [3]. In particular, natural polymers like polysaccharides provide several advantages such as biodegradability, biocompatibility, inexpensiveness, high availability, and biologically identifiable structural units that favor cellular activity in contrast to synthetic polymers [4]. Moreover, when applied as drug carriers, they can prevent the degradation of drugs, improve the drugs’ pharmacokinetic and pharmacodynamic profile, as well as increase the degree of absorption and penetration at specific target tissues [5]. Within the vast array of accessible polysaccharides, chitosan constitutes an ideal candidate for use in drug delivery systems since it has been proven to be non-toxic, biocompatible, biodegradable, low immunogenic, and bioadhesive [5]. A considerable amount of scientific effort has been devoted towards the study of chitosan-based materials intended for bioapplications relevant to drug delivery, and the obtained results have been the focus of several recent reviews [6,7,8,9,10,11].

Chitosan is a linear polysaccharide derived from the *N*-deacetylation of chitin, the second most abundant naturally occurring biopolymer after cellulose [3,7]. Chitin is present in the exoskeleton of crustaceans (i.e., crabs, lobsters, squids, and shrimps), insects, and fungal strains [5], and has a rigid crystalline structure [11]. The alkaline deacetylation of hard, inelastic chitin produces the random cationic copolymer chitosan, which is composed of *N*-acetyl-2-amino-2-deoxy-D-glucose units linked through β-(1,4) glycoside linkage [11]. Nevertheless, chitosan contains some residual acetamide groups, as determined by the degree of deacetylation (DD), which affects its overall physicochemical properties and especially its solubility [10]. Although chitosan has a low solubility at physiological pH values, in dilute acidic solutions, the primary amino groups are protonated and hence it becomes positively charged and water-soluble [5]. The positive charge of chitosan is also responsible for its advantageous properties related to anti-microbial activity, since it enables the interaction with negatively charged cell membranes of micro-organism. Accordingly, chitosan’s charges cause its mucoadhesive ability, which is attributed to the electrostatic interactions between the positively charged amino groups of the polymer chain with the negatively charged mucin glycoproteins residues, which are rich in sialic and sulfonic acids [5]. Moreover, due to its hemostatic properties and structural resemblance to glycosaminoglycans, such as hyaluronic acid, chitosan is considered a suitable candidate for wound healing applications [4]. Owing to these special characteristics, chitosan-based carriers have been readily employed for the delivery of proteins, peptides, growth factors, anti-inflammatory drugs, antibiotics, anticancer drugs, and vaccines, among others, as well as in gene therapy [5].

Despite numerous advantageous properties, chitosan’s low thermal resistance and surface area, along with its poor solubility in neutral and basic solvents, somehow limit its usage [11]. For this reason, physical or chemical modification of chitosan is essential in order to overcome any possible drawbacks and enhance its application. Physical modification usually entails the mixing and/or blending of two or more polymers, whereas chemical modification consists of grafting, crosslinking, and ionic gelation [3,6,11,12]. To this end, the presence of the amino and hydroxyl functional groups is really beneficial, since it facilitates the modification or derivatization of chitosan. Based on the specific reaction conditions, processes such as etherification, esterification, crosslinking, graft copolymerization, and *O*-acetylation are performed on hydroxyl groups, whereas reactions like acetylation, quaternization, Schiff’s base reaction, and grafting occur on amino groups [3]. Moreover, the positively charged amino groups and the high charge density of chitosan in acidic conditions enable the spontaneous formation of polyelectrolyte complexes with anionic polymers [13,14,15]. Polyelectrolyte complexation is regarded as a reversible physical crosslinking process without the use of chemical crosslinking agents or organic solvents, thereby reducing the potential toxicity and undesirable effects of the resulting complexed materials [13]. Furthermore, interpolyelectrolyte complexes have been proven to be successful in the solubilization of various hydrophobic drugs like quercetin [16], doxorubicin [17], and curcumin [18], thus demonstrating their suitability as drug delivery systems.

Overall, through the appropriate modification of chitosan, its physical and chemical properties (i.e., biocompatibility, bioactivity, biodegradability, and non-toxicity) can be improved, while at the same time its unique characteristics (antibacterial, anticancer, antiviral, etc.) are retained, thus expanding the application range of chitosan derivatives [8]. Along these lines, the use of polymers with extra functionalities for the modification of chitosan can lead to the formation of products that combine the innate properties and characteristics of the polysaccharide backbone with the ability to respond to external stimulus. One such intriguing example is the thermoresponsive poly(*N*-isopropylacrylamide) (PNIPAM), and several experimental studies have been devoted to the investigation of chitosan grafted with PNIPAM copolymers [19,20,21,22,23]. Quite interestingly, while PNIPAM is water soluble at room temperature, upon heating above its specific low critical solution temperature (LCST), which is about 32 °C, a reversible coil-to-globule phase transition occurs [19]. The transition from a hydrated/hydrophilic state with an expanded structure to a shrunken dehydrated state and collapsed structure is attributed to the disruption of hydrogen bonds with water molecules and the increase in interchain aggregation due to the prevalence of hydrophobic interactions. Most importantly, PNIPAM is deemed ideal for use in biorelevant applications like drug delivery systems, since its LCST is close to body temperature. More specifically, the temperature-induced increase in hydrophobic interactions is expected to improve cell adhesion, thus increasing cellular uptake [19].

The present study aims to investigate the formation of electrostatic complexes between two chitosan samples of distinct lengths (i.e., long versus short) and two end-functionalized PNIPAM homopolymers with different molecular weights, bearing a chargeable carboxyl group at one end. The electrostatic interaction between the positive amino groups of chitosan and the negative carboxylate end groups of the modified synthetic polymer is expected to result in the electrostatic binding of thermosensitive PNIPAM side chains onto the polysaccharide backbone. Therefore, it can be envisioned as a straightforward and facile method of producing chitosan–PNIPAM hybrid nanostructures that combine the properties of the two individual components and could even serve as an alternative to the chemical grafting method. Our main goal was the development of stable complexes that can be potentially used as drug delivery systems and the investigation of their physicochemical properties, especially in regard to the effect of the components’ molecular weight/length. The overall characteristics relative to the mass, size, size distribution, and effective charge of the complexes formed at different mixing ratios between the two components were investigated utilizing dynamic and electrophoretic light scattering (DLS and ELS) techniques. Moreover, polyelectrolyte titration provided additional information related to the charge density of the resulting complexes. Further insight into the complexes’ morphology and structure was obtained by scanning transmission electron (STEM) and atomic force (AFM) microscopy measurements. The hydrophobicity of the formed hybrid nanostructures was examined by fluorescence spectroscopy with the aid of a pyrene probe, both at ambient conditions and above the PNIPAM’s LCST. Furthermore, their response to changes in temperature as well as their stability against the increase in the ionic strength of the solution were explored by monitoring changes in their mass and size through DLS. Finally, the ability of these natural–synthetic hybrid macromolecular nanostructures to serve as potential drug delivery carriers was assessed for a representative Chi/PNIPAM complex system by utilizing curcumin as a model hydrophobic drug at different loading contents and examining the properties of the consequent drug-loaded complexes.

## 2. Materials and Methods

### 2.1. Materials

Two water-soluble chitosans (Chi) with different molecular weights and high deacetylation degrees (DD), Chi162K with *M*_W_ = 162 kDa and DD = 88.2%, and Chi1.5K with *M*_W_ = 1.46 kDa and DD = 90.2%, respectively, were provided by Shandong AK Biotech Co Ltd. (Jinan, China). The water solubility of these Chi samples is attributed to the presence of lactate and phosphate counterions, as evidenced by previous ^1^H-NMR measurements [24]. According to the *M*_W_ and DD values of each sample and the fact that the glucosamine and *N*-acetylglucosamine units have molecular weights equal to 161.2 and 203.2 Da [25], respectively, it was calculated that Chi162K has approximately 975 monomeric units, of which 860 deacetylated, while Chi1.5K has 9 monomeric units, of which 8 deacetylated. In parallel, two end-functionalized PNIPAM homopolymers with different molecular weights, specifically PNIPAM19K with *M*_W_ = 19,340 g/mol and PNIPAM3K with *M*_W_ = 3200 g/mol, as measured by size exclusion chromatography (SEC), were synthesized by reversible addition–fragmentation chain transfer (RAFT) polymerization using a previously published protocol [24]. Briefly, the reaction took place in dioxane medium at 70 °C for 6 h, using 4-cyano-4-((dodecylsulfanylthiocarbonyl)sulfanyl) pentanoic acid as the chain transfer agent (CTA) and 2,2-azobis(isobutyronitrile) (AIBN) as the initiator of the polymerization reaction. The resultant homopolymers were separated and purified using double precipitation in excess n-hexane. As a result of the specific synthetic route, the final PNIPAM chains had a dodecyl group at one end and a carboxyl at the other. All the necessary reagents used for the synthesis of the PNIPAM samples, as well as curcumin, were obtained from Sigma-Aldrich (Sigma Chemical Co., St. Louis, MO, USA).

### 2.2. Preparation of Chi/PNIPAM Complexes

Initially, stock solutions in ultrapure water with a concentration of 1 mg/mL were prepared for all four macromolecular samples, Chi162K, Chi1.5K, PNIPAM19K, and PNIPAM3K, and left to stand overnight for equilibration. In the PNIPAM stock solutions, appropriate aliquots of a 0.1 M NaOH solution in a final molar concentration equal to that of PNIPAM were added in order to ensure the transformation of all COOH groups into COONa. The pH of all stock solutions was measured as about 6. All stock solutions were filtered through 0.45 μm hydrophilic PET syringe filters (Chromafil, Macherey-Nagel, Düren, Germany) to remove any dust particles and impurities. Subsequently, the complexes were prepared by adding different volumes of the synthetic polymer solution, specifically 1, 2, and 4 mL, over 4 mL of the chitosan solution under magnetic stirring (~500 rpm). After 5 min, the final volume was adjusted to 10 mL by adding ultrapure water. In this way, all samples had the same final concentration of Chi equal to 0.4 mg/mL and varying PNIPAM concentrations, that is 0.1, 0.2, and 0.4 mg/mL. The pH of all complex solutions/dispersions was also measured as about 6. The complexes were kept for 24 h at room temperature before characterization. Table 1 summarizes the sample codes (where Comp stands for complex, L and S stand for long and short, referring to the length of the chitosan sample, and 1 and 2 indicate the two respective PNIPAM samples), the corresponding volume and molar ratios, as well as the calculated nominal charge ratios (assuming 1 positive charge per Chi deacetylated monomeric unit and 1 negative charge per PNIPAM chain) for each complex. Molar and charge ratios give an estimation of the relative amounts of the two macromolecular components for each system.

The stability of the formed complexes against the increase in ionic strength was also examined by titrating 1 mL of representative complex solutions/dispersions of the Chi/PNIPAM systems with appropriate aliquots of a 1 M NaCl solution, covering a range of ionic strength up to 0.5 M. The titration was monitored by means of DLS measurements and was performed directly in the instrument sample cell. After each addition step, the system was allowed to equilibrate for 10 min before measurement.

### 2.3. Drug Loading

For the investigation of the potential of the Chi/PNIPAM complexes as drug delivery carriers, a representative complex system corresponding to the combination of the long chitosan (Chi162K) and the long PNIPAM19K samples at a volume mixing ratio of 4:4, that is CompL1_4/4, was chosen as the most stable one. The initial solution of the complex was prepared as described in Section 2.2 and subsequently three different drug loading contents were examined by adding 2.5, 5, and 10% *w*/*w* of curcumin (CUR) in relation to the total mass of the complex. Specifically, 0.1, 0.2, and 0.4 mL of a 1 mg/mL CUR solution in ethanol were added to 5 mL of the initial CompL1_4/4 solution. The addition of the CUR solution was performed under stirring at 40 °C utilizing a water bath (as seen in Figure S1a) so as to benefit from the phase transition of the PNIPAM chains to the hydrophobic state, which was expected to enhance the interaction with CUR [26]. The mixture was left stirring at 40 °C for 15 min in order to ensure the incorporation of CUR and, as a next step, ethanol was evaporated by heating at 80 °C for 1.5 h. After EtOH evaporation and adjustment of the final volume to 5 mL with the addition of ultrapure water (in order to restore the amount of evaporated water), stable solutions/dispersions were obtained (Figure S1b). The sample codes, corresponding masses of the constituents, the nominal drug concentration, and the total volume for each solution are summarized in Table S1. The actual concentration of encapsulated CUR in the final solutions was determined utilizing a calibration curve of CUR in ethanol constructed by UV–Vis spectroscopy (SPECORD 200 Plus, Analytik Jena GmbH + Co. KG, Jena, Germany), as shown in Figure S2. Finally, the encapsulation efficiency, EE%, for each CUR loading content was calculated according to the following equation:

EE% = [concentration of encapsulated CUR/concentration of added CUR] × 100%.(1)

### 2.4. Dynamic and Electrophoretic Light Scattering

Valuable information about the scattered light intensity, the particle size, and the size distribution of the various Chi/PNIPAM complexes were obtained by means of DLS measurements. A Zetasizer Nano ZS (Malvern Instruments Ltd., Malvern, UK) equipped with a 4 mW He-Ne laser operating at a 633 nm wavelength and the Litesizer 500 device from Anton Paar (Graz, Austria) equipped with a 40 mW single-frequency laser diode operating at a 658 nm wavelength were employed. Measurements were performed at a scattering angle of 90°, either at ambient conditions or as a function of temperature. For the temperature-dependent measurements, the temperature was raised from 25 to 45 °C in 5 °C increments, followed by cooling of the samples back to 25 °C (before each measurement, a 10 min equilibration period was allowed). All obtained values were derived from mean measurements usually averaging 5 consecutive runs of 10 s. In a similar manner, ELS measurements were conducted on the same instrument at a 175° scattering angle in order to obtain the zeta potential of the complexes. It should be noted that for the measured scattered intensity values, there was a standard deviation of approximately 1–2%, while for the calculated hydrodynamic diameter and zeta potential values, the corresponding standard deviation was about 5%.

### 2.5. Polyelectrolyte Titration

The charge density of the formed Chi/PNIPAM complexes was determined using a PCD 03 particle charge detector (Mütek GmbH, Neckartailfingen, Germany). For this, 1 mL of the solutions/dispersions of the complexes was titrated using a strong polyanion, namely poly(ethylene sulfonic acid sodium salt) (PESNa, 0.87 mM). Specifically, the initial complex solution was first titrated with PESNa, followed by the ionization of the residual charges with the addition of a small aliquot of HCl 1 M and a further titration of the remaining charges with PESNa. From the total volume of polyelectrolyte solution needed for the full titration of each of the Chi/PNIPAM complexes (listed in Table S2), the milliequivalent charges (meq) were calculated and, according to the corresponding total mass of the complexes, their charge density was derived, expressed as meq of charges per g of sample (see also Table S2).

### 2.6. Scanning Transmission Electron Microscopy

The morphology of the formed complexes was evidenced by scanning transmission electron microscopy (STEM) using a Verios G4 UC (Thermo Fisher Scientific, Waltham, MA, USA) microscope. STEM investigations were performed in High Vacuum mode using the retractable specific detector STEM 3+ working at 20 kV. The complexes were deposited on 300 mesh copper grids coated with lacey carbon film. The obtained images were analyzed using the ImageJ software (version 1.54).

### 2.7. Atomic Force Microscopy

The resulting nanostructures were also analyzed by AFM, using an NTEGRA Spectra Atomic Force Microscope (NT-MDT Spectrum Instruments, Moscow, Russia) with commercially available silicon nitride cantilevers (NSG10, NT-MDT Spectrum Instruments, Moscow, Russia) in order to further evaluate their morphology and size. For this purpose, the complexes were deposited dropwise on freshly cleaved mica wafers and dried at an ambient temperature. Squares of 3 and 3 µm per side were scanned in the tapping mode in air.

### 2.8. Fluorescence Spectroscopy

Additional insight about the morphology of the Chi/PNIPAM complexes was gained by means of fluorescence spectroscopy measurements utilizing pyrene as a probe. In more detail, pyrene was added to a final concentration of 1 µM in the solutions/dispersions of the complexes. Utilizing an excitation wavelength of *λ* = 335 nm, the pyrene emission spectrum was obtained in the 340–600 nm range and the ratio of the intensities of the first peak *I*_1_ to the third peak *I*_3_ (located at about 372 and 383 nm, respectively) was calculated, in order to investigate the presence of hydrophobic regions in the formed complexes [27,28,29]. The spectra were recorded with the FLS980 Edinburgh Instruments (Livingston, UK) photoluminescence spectrometer, first at room temperature and subsequently at 45 °C, so as to examine possible changes in the microenvironment of the pyrene probe due to the phase transition of the PNIPAM chains.

## 3. Results

### 3.1. Physicochemical Properties of the Chi/PNIPAM Complexes

**Figure 1:**
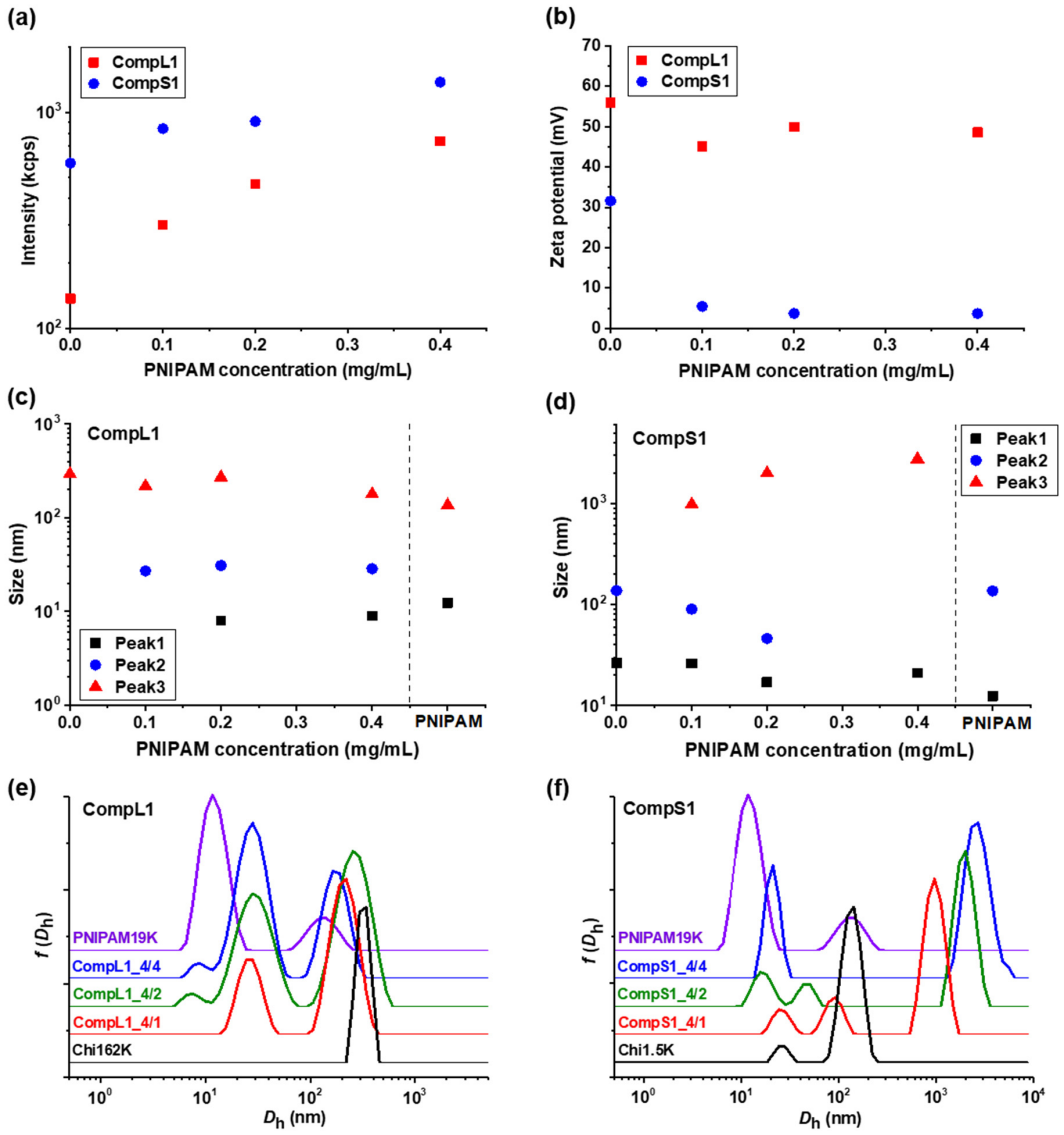
(**a**) Scattered intensity, (**b**) zeta potential, and (**c**,**d**) peak sizes, derived from the corresponding (**e**,**f**) size distributions, for the CompL1 and CompS1 series of complexes. Also included for comparison, the size distributions for the Chi162K, Chi1.5K, and PNIPAM19K samples.

Initially, the physicochemical properties of the obtained complexes between the long or short chitosan and the PNIPAM19K sample (i.e., CompL1 or S1 samples) were assessed by DLS and ELS measurements. The scattered intensity, zeta potential, peak sizes, and size distributions for the two series of complexes with different PNIPAM contents were analyzed and the obtained results are presented in Figure 1 as a function of the concentration of PNIPAM. It should be noted that the points at zero PNIPAM concentration indicate the corresponding values for the respective chitosan samples, that is Chi162K or Chi1.5K. Moreover, the sizes for the PNIPAM19K samples are also included in Figure 1c,d, separated by a vertical dashed line, while the size distributions of the corresponding initial components are also shown in Figure 1e,f for comparison. Finally, the corresponding polydispersity index values are shown in Figure S3a. As can be observed in Figure 1a, the scattered intensity for the CompL1 series gradually increased with increasing PNIPAM concentration. Since the scattered intensity was proportional to the mass of the complexes in solution, the observed increase confirms that the complexation between the two components occurred, and the mass of the complexes actually increased as more PNIPAM was added. In parallel, the scattered intensity for the CompS1 series also exhibited a similar increasing trend but with overall higher values, suggesting that the particles formed in this case had a greater mass or a denser conformation. This seems to be in direct correlation to the markedly higher initial scattered intensity of the Chi1.5K sample (point at 0 PNIPAM concentration), which implies that the short chitosan forms in solution particles of increased mass or density.

Regarding the effective charge of the complexes, the measured zeta potential values are presented in Figure 1b for both CompL1 and CompS1 series. Obviously, although no significant changes were observed upon increasing the concentration of PNIPAM for both series, the values of the zeta potential of the complexes were considerably lower than that of the corresponding chitosan samples, thus confirming the complexation between the two macromolecular components. This decrease was distinctly more pronounced in the case of the CompS1 samples, with the zeta potential values of the complexes being close to zero (which is also in agreement with the corresponding charge density values presented in Table S2), denoting the extended neutralization of opposite charges. In other words, the effective charge of the complexes suggests a higher degree of interaction (and thus, charge neutralization) between the short chitosan and the PNIPAM19K sample, as compared to that for the long chitosan, although the nominal charge ratio (that is, analogy of total number of charges) was similar for both series of samples (CompL1 or S1, see Table 1). This behavior can be ascribed to the possible difference in conformation of the two chitosan chains in solution, which was a direct consequence of the corresponding chain lengths of the two samples. In more detail, even in this case of semi-rigid chitosan chains, the longer one (Chi162K) was expected to adopt a somewhat coiled conformation, since its total chain length was rather high (i.e., 975 units × 0.52 nm/unit = 507 nm) in comparison to the persistence length of chitosans with high DD, which was found to be about 9 nm, thus exhibiting partial flexibility [30]. Therefore, the charges inside the coil were less available for the subsequent electrostatic interaction with the –COO^−^ end groups of the PNIPAM chains, mainly after some chitosan/PNIPAM interchain interactions had occurred. On the other hand, the conformation of the short chitosan was expected to be a more extended one, as its chain length was significantly smaller (i.e., 9 units × 0.52 nm/unit = 4.7 nm). This in turn allowed the charges from the amino groups to be more accessible for interaction with the carboxyl PNIPAM chain ends. Furthermore, the fact that, in both cases, the increase in the PNIPAM concentration did not have a significant effect on the overall charge of the formed complexes can also be attributed to their different conformation/structure. It is rather likely that the assumed less compact/more loose conformation of the ones formed with the long chitosan allowed for secondary conformational rearrangements (in an attempt to maintain colloidal stability), which led to the observed preservation of the overall positive charge.

As far as the size of the complexes is concerned, from Figure 1c,e it can be observed that for the CompL1 series, two main peaks (Peak 2 and 3) were discerned, with their size (i.e., about 30 and 220 nm, respectively) being more or less the same irrespective of the PNIPAM concentration in the complexes. These peaks denote the presence of two different scattering populations in the solutions/dispersions of the complexes, which most probably correspond to particles comprising different numbers of complexed chains. It is possible that the long chitosan exhibited some degree of self-assembly in the solution, forming multi-chain aggregates comprising a small number of chains, due to hydrophobic interactions stemming from the hydrophobicity of its backbone and pendant acetate side groups. This assumption seemed to be confirmed by the corresponding size distribution of the Chi162K sample, which showed a main peak at about 300 nm (Peak 3). Even though peaks corresponding to smaller sizes were not detected, this size fits better to the hypothesis of multi-chain aggregates rather than single chains. Therefore, the two observed populations of complexes could be the result of the interaction of either single chains or multi-chain aggregates of chitosan with the PNIPAM polymer, with the latter exhibiting some shrinking upon complexation, as evidenced by the corresponding reduction in size. Nevertheless, they seemed to adopt a rather open/loose conformation/structure (mostly dictated by that of the corresponding species of chitosan), since their sizes were not significantly affected by the interaction with an increasing number of PNIPAM chains. A small peak at about 10 nm (Peak 1) was also detected for the CompL1_4/2 and 4/4 samples and could indicate the presence of uncomplexed synthetic polymer chains, since it coincided with the corresponding peak of the PNIPAM19K sample. Another interesting remark about the PNIPAM19K sample is that it also exhibited a small degree of self-aggregation (Peak 3), likely due to hydrophobic interchain interactions (which were further enhanced by the dodecyl end groups in this case).

For the CompS1 series of complexes, the short chitosan also exhibited self-assembly in solution, since two peaks (Peak 1 and 2) with sizes about 25 and 150 nm, most likely corresponding to single chains and multi-chain aggregates, are seen in Figure 1f. At the same time, the size distributions of the CompS1_4/1 and 4/2 samples showed three peaks, with the size of the first two being somewhat smaller than that of the corresponding peaks of the Chi1.5K sample. Apparently, both the single chains and the multi-chain aggregates of the short chitosan interacted electrostatically with the PNIPAM chains, forming complexes that were slightly smaller than the initial species of chitosan as a result of the reduction in electrostatic repulsions due to charge neutralization and/or the increase in hydrophobic interactions caused by the incorporation of PNIPAM. This combination of effects clearly weakened the colloidal stability of the formed complexes, eventually leading to extended secondary aggregation, as evidenced by the presence of the much larger population corresponding to Peak 3 with sizes almost equal or even greater than 1 μm. In fact, this secondary aggregation became more pronounced as the PNIPAM concentration increased, as the size of the largest population gradually increased, and the two smaller populations seemed to merge into one in the case of CompS1_4/4 sample. By also considering the fact that no free/uncomplexed PNIPAM chains appeared to be detected (contrary to the CompL1 series), it can be assumed that the short chitosan enabled a greater degree of interaction with the PNIPAM chains compared to the long one, as previously discussed. Of course, this could be related to the shortest polysaccharide molecule that allowed for a higher accessibility of its charges, in contrast to the longer one. One final remark concerning both CompL1 and S1 series is that the presence of the different scattering populations in solution also entailed a rather high polydispersity in all cases (see Figure S3 and corresponding discussion).

**Figure 2:**
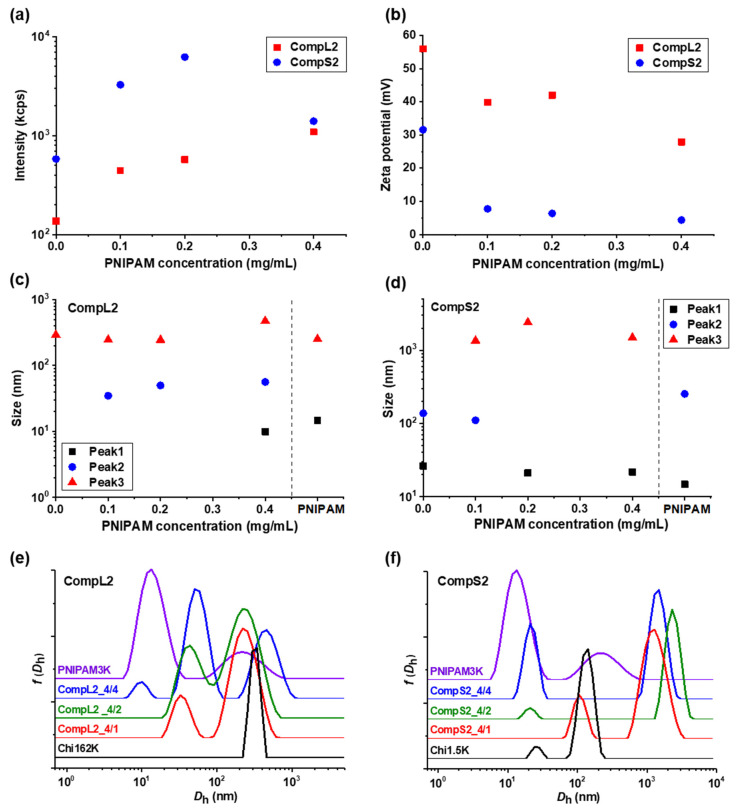
(**a**) Scattered intensity, (**b**) zeta potential, and (**c**,**d**) peak sizes, derived from the corresponding (**e**,**f**) size distributions, for the CompL2 and CompS2 series of complexes. Also included for comparison, the size distributions for the Chi162K, Chi1.5K, and PNIPAM3K samples.

The solution behavior of the hybrid nanostructures formed between both chitosans and PNIPAM3K (i.e., CompLorS2 samples) was investigated in a similar manner by DLS and ELS measurements, and the overall physicochemical characteristics in regard to mass, charge, size, and size distribution are presented in Figure 2, while the polydispersity index is shown in Figure S3b. It is quite obvious that the obtained results show a similar trend to the ones already discussed for the corresponding complexes with the PNIPAM19K sample. Specifically, in Figure 2a, an analogous increase in the scattering intensity as a function of PNIPAM concentration was observed for both series of complexes, verifying the complexation of both the long and the short polysaccharide molecules with the synthetic polymer chains. Once again, the mass of the complexes corresponding to Chi1.5K was higher than the ones for Chi162K, and, at the same time, significantly higher than that of the CompS1 series, demonstrating an increased interaction between the short chitosan and the PNIPAM3K chains. This seems to be in agreement with the fact that for the PNIPAM3K sample, the number of chains corresponding to each chitosan molecule was larger (see Table 1), which apparently further promoted secondary aggregation. The observed decrease in intensity for the CompS2 series at the highest PNIPAM content could possibly be explained by some small amount of precipitation, also indicative of the stronger interaction. Moreover, the zeta potential measurements (Figure 2b) show lower values for the complexes compared to the corresponding pure chitosan samples, especially for the CompS2 series. This observation is consistent with the notion that the shorter chitosan molecule was able to more easily interact with PNIPAM chains than the longer one, and hence the charge neutralization was more extended in this case, with the effective charge of these complexes being close to zero. Regarding the size of the complexes, the distributions of Figure 2e,f reveal two main populations for the CompL2 series, corresponding to the complexes formed between the single chain and multi-chain aggregates of chitosan with the PNIPAM chains, while an additional (third) larger population indicative of secondary aggregation was observed for the CompS2 complexes. For both CompLorS2 series, the sizes of the individual peaks, as well as their variation upon increasing the concentration of PNIPAM, were quite similar to those witnessed for the CompLorS1 complexes. This fact suggests that in regard to the size of the complexes, the determining factor was mainly the conformation/self-aggregation of the chitosan molecules and, to a much lesser extent, the length of the PNIPAM chains. As in the previous case, the solutions exhibited increased polydispersity as a consequence of the coexistence of various populations (see Figure S3b).

**Scheme 1:**
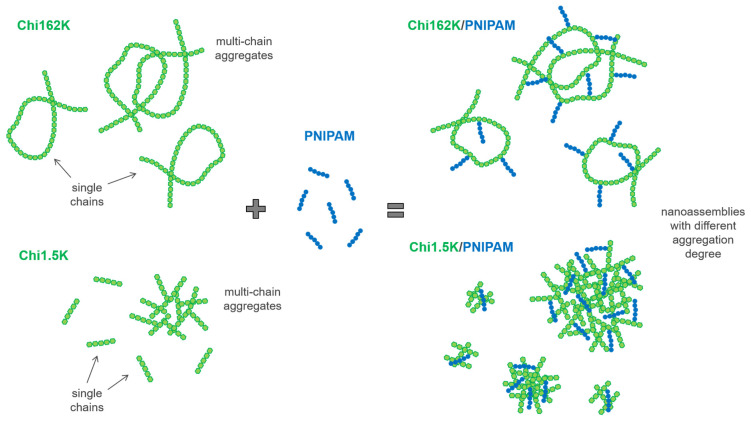
Schematic illustration of the proposed complexation between the two chitosan samples (i.e., Chi162K and Chi1.5K) and end-functional PNIPAM chains (independent of length), depicting the different types of formed nanoassemblies (components are not drawn exactly to scale).

Overall, the combination of DLS and ELS results suggest that for all four investigated Chi/PNIPAM systems, different types of nanoassemblies with varying degrees of complexation/aggregation coexisted in the solution. Their respective structures seemed to be dictated mainly by the conformation of the corresponding chitosan samples, regardless of the PNIPAM chain length. A schematic representation of the complexation process between the two chitosan samples and PNIPAM chains (independent of length), along with the proposed structure of the resulting complexes/aggregates, is given in Scheme 1.

Another interesting characteristic of the formed Chi/PNIPAM complexes is their charge density, which was indirectly determined by means of polyelectrolyte titration experiments, and the obtained values are given in Table 2 (for the corresponding calculations see Table S2). The polyelectrolyte titration method determined the excess charges of the complexes by neutralizing them with an oppositely charged strong polyelectrolyte. As expected, for all four series of complexes, the charge density exhibited the highest values for the samples corresponding to the 4/1 volume ratio, since these systems had the higher amounts of chitosan in excess. By increasing the PNIPAM content in the complexes, the charge density gradually decreased in all cases, as a result of the occurring charge neutralization due to the electrostatic complexation between the positive amino groups of chitosan and the negative carboxylic end groups of PNIPAM. In parallel, the complexes formed with the long chitosan (CompL1or2) had a higher charge density than the ones corresponding to the short polysaccharide molecule (CompS1or2), which exhibited a charge density close to zero (in agreement with the results obtained for zeta potential included in Figure 1b). This finding corroborates the concept that the significantly shorter Chi1.5K molecule allows for a higher degree of interaction with the PNIPAM chains, leading to extended charge neutralization. Furthermore, as far as the effect of the length of the PNIPAM chain on the charge density of the formed complexes is concerned, the complexes corresponding to the shorter sample exhibited slightly lower values than those for the longer one. This observation seems to be in agreement with the fact that for the same volume ratio, more PNIPAM3K chains corresponded to each chitosan molecule, in comparison to the PNIPAM19K sample, as expressed by the molar ratio of the two components shown in Table 1.

Additional information relevant to the hydrophobicity of the Chi/PNIPAM complexes was derived through fluorescence spectroscopy measurements with the aid of the pyrene probe, which has a distinctive emission spectrum indicative of the polarity of its microenvironment [27,28,29]. The acquired fluorescence spectra both at 25 and 45 °C for all the series of complexes are shown in Figure S4, while Table 3 presents the corresponding values of the characteristic *I*_1_/*I*_3_ intensity ratio. Under ambient conditions, the CompS1 series exhibited *I*_1_/*I*_3_ ratio values of about 1.2, which indicate a fairly hydrophobic microenvironment, while the other three series of complexes showed *I*_1_/*I*_3_ ratio values of about 1.6, which corresponds to a rather hydrophilic one. It seems that in the case of the complexes formed between the long chitosan and either of the two PNIPAM samples, their more open/loose conformation allowed for a greater degree of hydration. On the other hand, the extended interaction and consequent secondary aggregation occurring for the CompS1 series, led to the formation of denser nanostructures with a more compact/collapsed conformation, and thus increased hydrophobicity. Quite interestingly, for the CompS2 samples, the corresponding *I*_1_/*I*_3_ ratio values suggest that the pyrene probe experienced a hydrophilic microenvironment, but, at the same time, excimer formation indicative of close proximity between pyrene molecules was observed, as evidenced by the relative characteristic peak (see Figure S4). In other words, a coexistence of both isolated pyrene molecules in hydrophilic regions and hydrophobic domains/pockets, where the pyrene molecules aggregate due to increased crowding conditions, was detected. This could be correlated to the length of the PNIPAM chains, in the sense that the longer ones (i.e., PNIPAM19K sample) seemed to enable the formation of large hydrophobic regions, possibly acting as bridging elements between complexes, in contrast to the shorter ones (PNIPAM3K) that only allowed for local spatially confined hydrophobic domains.

**Figure 3:**
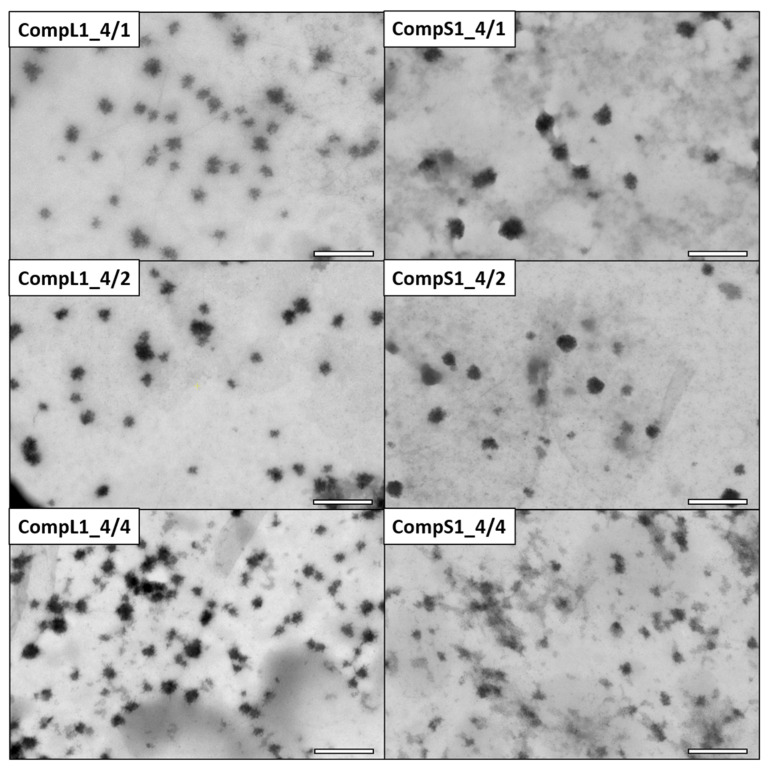
STEM images for the CompL1 (**left**) and CompS1 (**right**) series of Chi/PNIPAM complexes. The scale bar corresponds to 200 nm.

**Figure 4:**
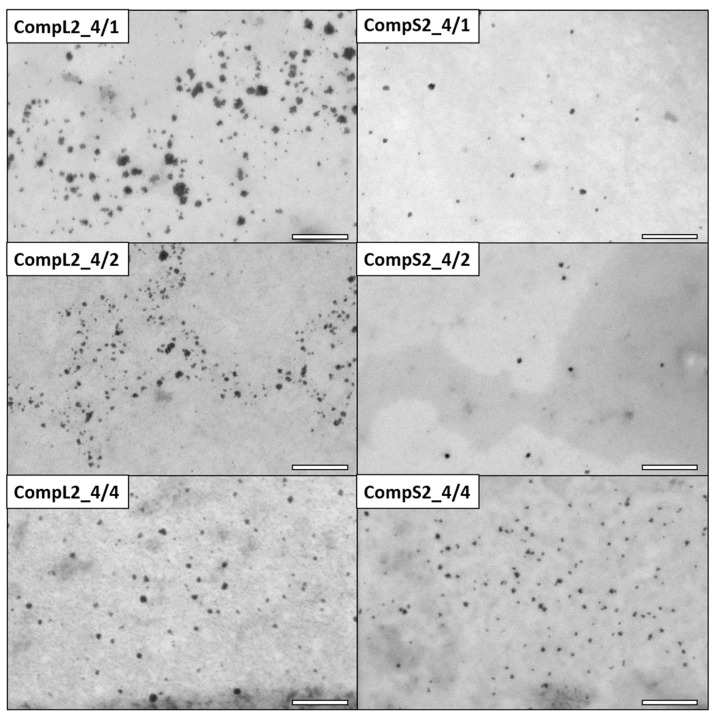
STEM images for the CompL2 (**left**) and CompS2 (**right**) series of Chi/PNIPAM complexes. The scale bar corresponds to 200 nm.

The morphology of the various Chi/PNIPAM complexes was investigated by STEM, and the obtained images for the CompLorS1 and CompLorS2 series are respectively shown in Figure 3 and Figure 4. In all cases, rather spherical homogeneous particles were discerned, while upon closer inspection it can be observed that the nanoparticles show an urchin-like morphology, most probably stemming from the partial rigidity of the chitosan backbone. Due to its linear structure and multitude of available functional amino groups, each chitosan chain was expected to electrostatically interact with more than one PNIPAM chain (since the synthetic polymer has only one charged end group per chain), thus creating primary complexes whose morphology would mostly be dictated by that of the conformation and aggregation state of the chitosan macromolecules. As a result of the reduction of the electrostatic repulsions due to charge neutralization that further favor the intra-chain interactions of chitosan and also reduce the overall solubility, the initial complexes aggregated together, rendering populations of clustered particles with larger sizes (see Figure S5). The average diameter of the nanoparticles was evaluated with the aid of ImageJ software by measuring all the individual particles found in each image. In this manner, the nanoparticles’ average diameter for the CompL1 series appeared to not vary significantly with the increase in PNIPAM, in accordance with the DLS observations, with all three complexes having an average size of about 33 nm. On the other hand, the average diameter for the CompS1 samples showed a slight decrease from about 45 nm (CompS1_4/1) to 26 nm (CompS1_4/4), indicating the formation of more compact structures as the PNIPAM concentration increased.

As far as the corresponding complexes formed between the two chitosan molecules and the PNIPAM3K sample (CompL2 or S2) are concerned, the STEM images of Figure 4 clearly show particles of smaller sizes compared with the ones containing PNIPAM19K (CompL1 or S1). In more detail, the average diameter of the nanoparticles for the CompL2 sample with the lowest amount of PNIPAM (CompL2_4/1) is about 19 nm, while the size further decreases to about 10 nm with the increase in PNIPAM concentration (CompL2_4/4). By comparison, CompS2 samples exhibited even smaller nanoparticle average diameters, ranging from about 10 nm (CompS2_4/1) to 7 nm (CompS2 4/4). Of course, it should be noted that in all cases the sizes calculated from the STEM images were expected to be smaller than the ones determined by DLS, because DLS analyzes particles in their swollen state in solution, while STEM technique requires the complete evaporation of the water, or, in other words, inspects their dry state. Therefore, although according to the DLS results the complexes corresponding to the two different PNIPAM samples seemed to have similar sizes, the fact that STEM images indicated a smaller size for the complexes formed with the PNIPAM3K chains is indicative of their more compact/dense structure. One final remark is that the particles seen in the STEM images of Figure 3 and Figure 4 are most probably primary complexes that correspond to the smaller in size populations discerned by DLS. Nevertheless, in some cases, larger clustered structures were also evidenced in STEM, as shown in Figure S5.

**Figure 5:**
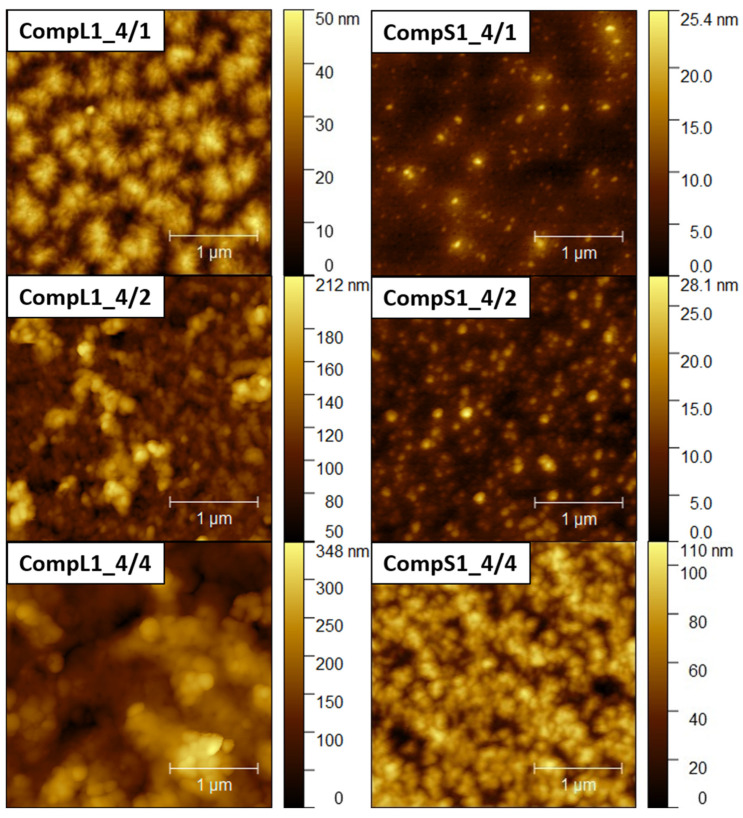
AFM images for the CompL1 (**left**) and CompS1 (**right**) series of Chi/PNIPAM complexes. The scale bar corresponds to 1 μm.

**Figure 6:**
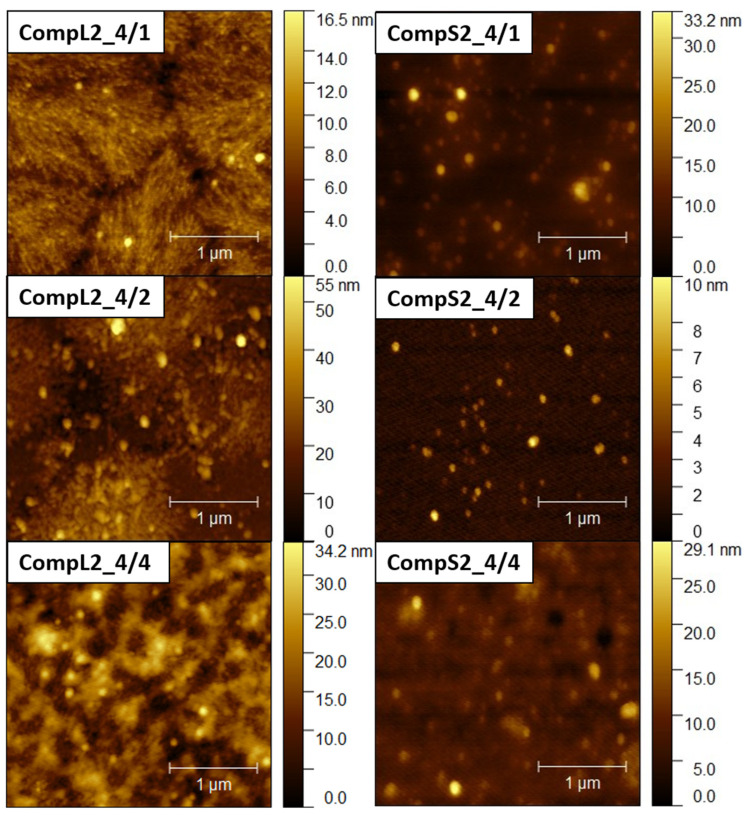
AFM images for the CompL2 (**left**) and CompS2 (**right**) series of Chi/PNIPAM complexes. The scale bar corresponds to 1 μm.

Further insight into the size and structure of the formed complexes was obtained through AFM imaging. Figure 5 and Figure 6 present representative AFM images for the CompLorS1 and CompL2 or S2 series, respectively, while the corresponding size measurements of individual surface profiles are shown in Figures S6–S9. From the analysis of the AFM images, it can be inferred that in all cases rather compact spherical particles, either sparsely distributed or closely packed together, were deposited on the mica substrate. For both the CompL1 and L2 series, the lowest PNIPAM content samples (CompL1 or L2_4/1) exhibited a rather interesting morphology, which could be indicative of the long chitosan conformation and the rigidity of the polysaccharide backbone. Furthermore, for all three CompL1 samples, the sizes of the individual particles were in the range of 100 to 250 nm (see Figure S6), suggesting that they corresponded to structures formed by the aggregation of primary complexes. A narrower distribution and relatively smaller sizes were observed for the CompS1 series, as all three samples showed particles with diameters in the range of 100 to 150 nm (Figure S7), which could be correlated with their assumed more compact/dense structure. This also stands for both the CompL2 and S2 series of samples, with the observed particles having sizes of about 100 nm (Figures S8 and S9). Note that smaller particles were discerned in most of the obtained AFM images, indicating the coexistence of various populations of complexes comprising different numbers of chitosan molecules and PNIPAM chains. Lastly, it was also interesting that the measured heights for the complexes of the long chitosan (CompL1 or L2) were generally larger than those corresponding to the short one (CompS1 or S2). This could indicate a reduced ability to adopt more flattened conformations upon surface contact/deposition, which might be a consequence of the longer length of the polysaccharide molecule.

### 3.2. Temperature Response of the Chi/PNIPAM Complexes

**Figure 7:**
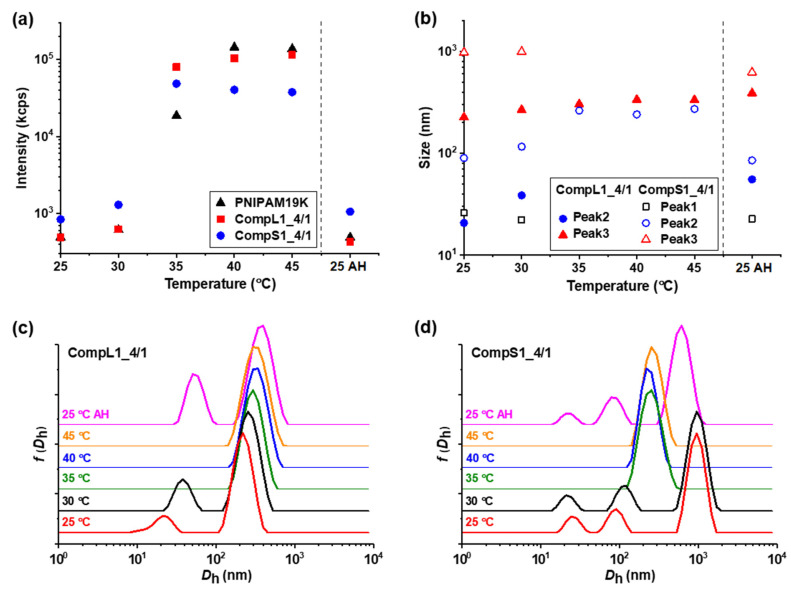
Temperature effect on the (**a**) scattered intensity, and (**b**) peak sizes, derived from the corresponding (**c**,**d**) size distributions, for the CompLorS1_4/1 samples.

**Figure 8:**
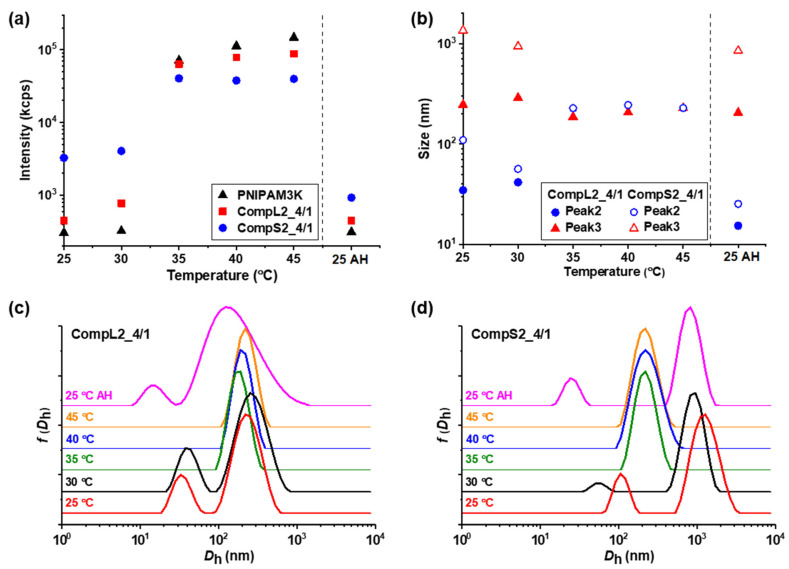
Temperature effect on the (**a**) scattered intensity, and (**b**) peak sizes, derived from the corresponding (**c**,**d**) size distributions, for the CompLorS2_4/1 samples.

Since PNIPAM is a thermoresponsive polymer, it was expected to impart the same characteristics to the Chi/PNIPAM complexes as well, and for this reason, the influence of temperature on the formed complexes was investigated. In practice, their thermal response was examined by performing DLS measurements at different temperatures between 25 and 45 °C (with 5 °C increment steps), and the obtained results regarding the scattered intensity, peak sizes, and corresponding size distributions for the representative CompLorS1_4/1 and CompLorS2_4/1 samples are shown in Figure 7 and Figure 8, respectively. The influence of temperature on the rest of the Chi/PNIPAM complexes and the two PNIPAM samples was also investigated and the results can be found in the Supplementary Materials (see Figures S10–S14). For all investigated samples, after heating up to 45 °C, the temperature was brought back to 25 °C and the sample was remeasured in order to examine the reversibility of the structures formed by the heating process. These results are denoted as “AH” (i.e., after heating) and are separated by a vertical dashed line in the graphs of scattered intensity and peak sizes.

Figure 7a and Figure 8a reveal the influence of temperature on the scattered intensity for the representative complexes of both chitosan samples at a 4/1 volume ratio in comparison to the corresponding PNIPAM samples. At 35 °C (i.e., above the LCST of PNIPAM) an abrupt increase in intensity accompanied by a significant increase in turbidity was observed for all samples. This behavior is characteristic of the thermal phase transition of PNIPAM, and was attributed to the increase in the hydrophobic interactions that induce interchain aggregation and was common for all investigated samples of Chi/PNIPAM complexes (see also Figures S10–S13a). Further increases in temperature up to 45 °C seemed to have no significant effect on the scattered intensity, while the initial values were fully restored upon returning to 25 °C, showcasing the reversibility of the transition. At the same time, the corresponding size distributions for the 4/1 complexes of all four systems (Figure 7c,d and Figure 8c,d) exhibited the same transition from two or three peaks to only one above 35 °C, which was also a characteristic trait of both PNIPAM samples (see Figure S14). This behavior suggests the aggregation of the initial populations of complexes into single compact structures, owing to the increase in the hydrophobicity of the complexed PNIPAM chains, which in turn favors interchain interactions. The sizes of these thermally induced aggregates seems to have some correlation to the conformation of the initial complexes. In particular, for the complexes of the long chitosan molecules (CompL1or2_4/1), the species detected above 35 °C had a similar size to the corresponding initial larger populations, while in the case of the short chitosan complexes, intermediate sizes (in comparison to the initial populations) were observed for the aggregates formed as a result of the thermal transition. This fact could mean that the looser conformation of the complexes formed with the long chitosan hindered, to some degree, the inter-chain PNIPAM interactions, thus retaining more or less their initial structure upon heating. On the contrary, the complexes of the short chitosan already had a more dense/compact conformation that facilitated to a greater extent the thermally induced PNIPAM interactions, leading to further increases in their compactness. Similar thermal transitions were observed for the rest of the Chi/PNIPAM complexes (see Figures S10–S13), with the only difference worth mentioning being those observed for the sizes of the CompLorS2_4/4 samples (Figure S13). In this case, the long chitosan complexes seemed to become more compact upon heating, while those of the short chitosan maintained their size. Apparently, as the PNIPAM content increased, the inter-chain interactions became more dominant and even the relatively loose initial (i.e., before heating) conformation of the CompL2 complexes collapsed, while the already too-compact structure of the CompS2 ones did not allow for additional reduction in their size. Nevertheless, after the solutions were cooled back to room temperature, they all returned to their initial state, again verifying the reversibility of the systems.

The enhancement of the overall hydrophobic interactions in the systems of the formed Chi/PNIPAM complexes upon increasing the temperature above the LCST of PNIPAM, was also confirmed through the pyrene fluorescence measurements performed at 45 °C. As seen in Table 3, the corresponding *I*_1_/*I*_3_ ratios exhibited more or less lower values compared to the ones at ambient conditions in almost all cases, demonstrating an increase in the hydrophobicity of the microenvironment probed by pyrene. As already discussed, the innate conformation of both the complexes and their constituent macromolecules greatly influenced their structural/conformational thermal transition, and this was also evidenced through the changes in the *I*_1_/*I*_3_ ratios. In particular, the complexes containing the PNIPAM19K sample exhibited a somewhat larger decrease in the *I*_1_/*I*_3_ ratios that the ones formed with the PNIPAM3K, which is to be expected since the longer PNIPAM chains entailed more extensive inter-chain aggregation.

### 3.3. Ionic Strength Effect on the Chi/PNIPAM Complexes

**Figure 9:**
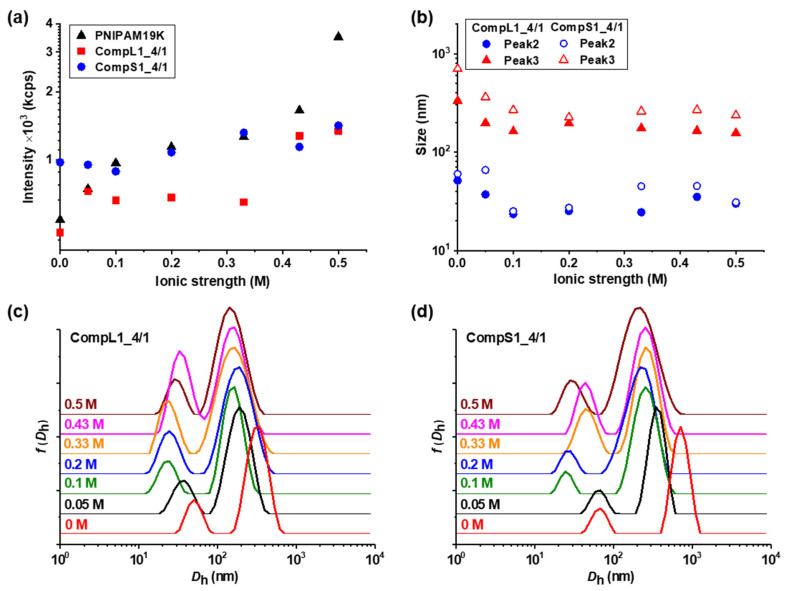
Ionic strength effect on the (**a**) scattered intensity and (**b**) peak sizes, derived from the corresponding (**c**,**d**) size distributions, for the CompLorS1_4/1 samples.

**Figure 10:**
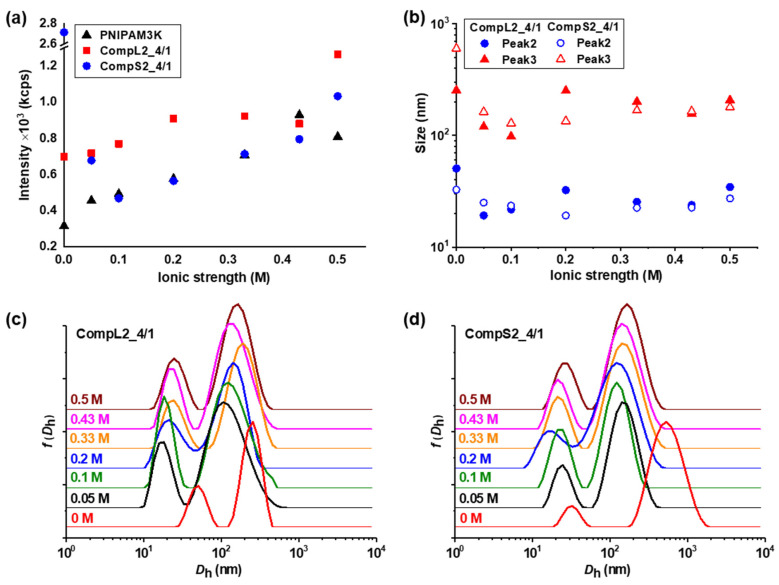
Ionic strength effect on the (**a**) scattered intensity, and (**b**) peak sizes, derived from the corresponding (**c**,**d**) size distributions, for the CompLorS2_4/1 samples.

Another external stimulus that can affect the properties and behavior of the various Chi/PNIPAM complexes under study is the increase in the ionic strength of the solution. In order to investigate this effect on the already formed complexes, relevant DLS measurements were performed and the results concerning the scattered intensity, the peak sizes, and the corresponding size distributions for the CompLorS1_4/1 and CompLorS2_4/1 samples were plotted against the ionic strength of the solution, as shown in Figure 9 and Figure 10. The ionic strength effect was also examined for the CompLorS1_4/4 and CompLorS2_4/4, as well as the two PNIPAM and the two chitosan samples, and the obtained results are presented in a similar manner in Figures S15–S18.

In general, Figure 9 and Figure 10 reveal analogous changes in both the intensity and the individual sizes for the complexes corresponding to the same PNIPAM sample. In more detail, CompL1or2_4/1 samples exhibited a slight gradual increase in intensity as a function of ionic strength, while the size of both populations decreased upon the initial addition of salt (0.05 M) up to 0.1 M, but it remained rather unaffected by the further increase in ionic strength. This combination of events could possibly mean that the complexes shrink or collapse as a result of the occurring charge screening and the parallel reduction in electrostatic repulsions brought on by the increase in ionic strength. In this way, a reduction in their size and, at the same time, an increase in their scattered intensity as they become more compact/dense (possibly followed by an increase in their mass) was observed. In parallel, CompS1or2_4/1 samples exhibited a comparable transition in sizes, while the scattered intensity initially (up to 0.1 M) decreased either slightly, in the case of CompS1, or significantly, for CompS2. Therefore, it can be assumed that for the complexes formed with the short chitosan molecules, the initial increase in ionic strength and the consequent charge screening resulted in some partial disassembly of the aggregated secondary complexes. Nevertheless, upon further increase in ionic strength (0.2–0.5 M), no significant changes were observed.

Similar effects on the scattered intensity and the corresponding sizes for both CompLorS1_4/4 and CompLorS2_4/4 samples, with an even more pronounced reduction in the size of the larger population for the CompS1or2_4/4 samples (i.e., indication of disassembly) were detected, as shown in Figures S15 and S16. The only observable difference was that the sizes of the two populations discerned for the CompL1_4/4 sample seemed practically unaffected by the increase in ionic strength. One probable explanation is that in this case, the presence of a larger number of PNIPAM chains (i.e., high PNIPAM concentration) in these complexes, in combination with the longer lengths of both the chitosan molecule and the PNIPAM19K sample, stabilized their structure more, in the sense that the conformation of complexed PNIPAM chains did not shrink/collapse due to steric effects from neighboring chains. Overall, it was established that the response of the formed complexes to the increase in the ionic strength of the solution was closely related to their initial conformation and structure. Moreover, it was rather interesting that the observed changes occurred at relatively low ionic strength values below 0.2 M, which also had a biological relevance (i.e., ionic strength was about 0.15 M for blood plasma or cell cytoplasm). In comparison, the two PNIPAM samples (Figure S17) showed a slight increase in both their scattered intensity and the sizes of their two populations (a little more pronounced for the PNIPAM3K sample), suggesting some small degree of secondary aggregation due to the phase transition of the PNIPAM chains to their collapsed state upon the increase in ionic strength [31,32,33]. The neutralization of charges also caused some small degree of secondary aggregation of the chitosan molecules (Figure S18), with the effect being more evident in the case of the short Chi1.5K sample. One final interesting remark is that for the long chitosan, additional peaks denoting the presence of smaller-in-size populations were observed as a result of the increase in ionic strength (Figure S18c), which could be attributed to a partial disassembly of the multi-chain aggregates of the Chi162K sample.

### 3.4. Drug Loading Capacity of the Chi/PNIPAM Complexes

The ability of the specific chitosan–PNIPAM hybrid nanostructures to serve as potential drug delivery systems was explored by examining their capacity to encapsulate a model hydrophobic drug, namely curcumin (CUR). Curcumin is widely known for its antioxidant, anti-inflammatory, antimicrobial, and anticancer properties [34], and, at the same time, it is a fluorescent molecule that can be used for bioimaging purposes [35,36]. Unfortunately, the low solubility of curcumin in water (below 6 μg/mL) restricts its broad applicability since it results in low cellular uptake, poor oral bioavailability, and low chemical stability [34]. One possible solution is the use of suitable polymeric carriers in an attempt to improve the bioavailability of curcumin and thus improve its effectiveness. Among the various Chi/PNIPAM complexes that were previously investigated, CompL1_4/4 was chosen for the encapsulation of CUR, as the combination of the long chitosan (Chi162K) molecule with the long PNIPAM19K chains is considered to be the most stable one, while the high PNIPAM content was expected to further enhance the interaction with the hydrophobic drug. Three different drug loading ratios were investigated by adding 2.5, 5, and 10% *w*/*w* of CUR in regard to the total mass of the complex, and the mixing was performed at 40 °C in order to benefit from the phase transition of the PNIPAM chains, as described in detail in Section 2.3.

**Figure 11:**
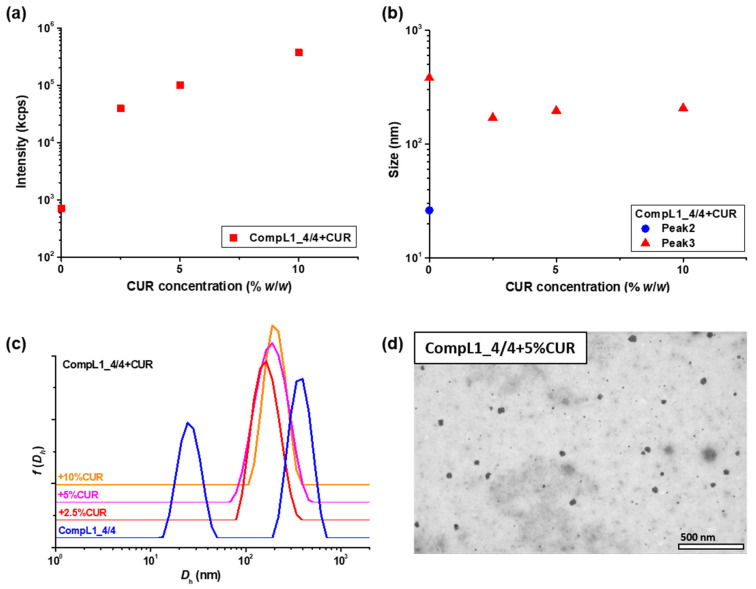
DLS results in regard to the (**a**) scattered intensity and (**b**) peak sizes, derived from the corresponding (**c**) size distributions, for the CUR-loaded CompL1_4/4 samples, along with (**d**) a representative STEM image for the CompL1_4/4 + 5%CUR sample (the scale bar corresponds to 500 nm).

The physicochemical properties of the resulting CUR-loaded CompL1_4/4 samples were investigated by performing DLS measurements on 1:10 diluted solutions, and Figure 11 presents the obtained scattered intensity values, and the peak sizes from the corresponding size distributions. Moreover, the morphology of the CUR-loaded nanoparticles was evaluated by STEM for the representative CompL1_4/4 + 5%CUR sample and a characteristic image is shown in Figure 11d. As can be seen, the DLS results reveal that the CUR-loaded complexes exhibited a drastic increase in scattered intensity, signifying an increase in the mass of the structures (Figure 11a). Meanwhile, as far as the size of the drug-loaded complexes is concerned, a transition from the two peaks discerned for the initial complex to one peak with an intermediate size was detected, which was accordingly accompanied by a parallel significant decrease in the polydispersity index (i.e., from about 0.6 to 0.1). The combination of the observed changes confirmed the successful incorporation of CUR and further suggested the shrinking of the initial complexes and the formation of more compact/dense structures, apparently as a result of the increase in hydrophobic interactions due to the presence of the CUR molecules. Still, the overall positive effective charge of the primary Chi/PNIPAM complexes was preserved, as the sample with the highest CUR loading content (i.e., CompL1_4/4 + 10%CUR) exhibited a zeta potential value of about +30 mV. Finally, the morphology of the drug-loaded nanostructures remained spherical and in close correlation to that of the initial complex, while their corresponding average size was about 40 nm. This was slightly higher than that derived from STEM for the initial complex (i.e., about 33 nm), which could also be indicative of the more dense/compact structures formed after CUR loading.

**Figure 12:**
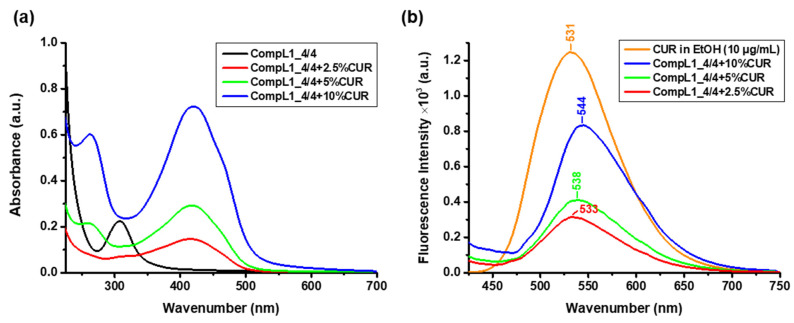
(**a**) UV–Vis and (**b**) fluorescence spectra for the CUR-loaded CompL1_4/4 samples.

In order to determine the concentration of the encapsulated CUR, UV–Vis spectroscopic measurements were performed for the same diluted solutions and the acquired spectra are shown in Figure 12a, along with the corresponding spectrum of the CompL1_4/4 for comparison. From the obtained values of the absorbance at 427 nm and the constructed calibration curve of CUR in ethanol (Figure S2), the actual concentration of encapsulated CUR in the solutions/dispersions of the complexes was derived. The corresponding values for each loading ratio are given in Table 4, along with the concentration of the added CUR (i.e., nominal concentration) and the encapsulation efficiency EE% was calculated according to Equation (1). The estimated EE% values were in the range of 43 to 61% for the three CUR contents investigated (i.e., 2.5, 5, and 10% *w*/*w*), indicating a sufficient drug loading capacity for the initial Chi162K + PNIPAM19K complex, which was undoubtedly correlated to the overall hydrophobicity of the system. Finally, fluorescence spectroscopy was employed to verify the fluorescent properties of the CUR-loaded complexes and thus their potential use in relevant bioimaging applications. Figure 12b presents the obtained spectra for the three different investigated CUR contents, along with the corresponding spectrum of pure CUR in ethanol (with a concentration of 10 μg/mL). In comparison to pure CUR, an apparent gradual red shift of the characteristic CUR peak from 531 to 544 nm can be seen for the drug-loaded complexes. This shift is attributed to the hydrophobic interactions of CUR with the PNIPAM chains (especially since the loading procedure was conducted above the LCST) [37,38], while it is possible that the hydrophobic regions of the chitosan backbone also facilitate CUR encapsulation. Nevertheless, all three formulations exhibited strong fluorescent properties.

## 4. Conclusions

The subject of this study was the construction of natural–synthetic macromolecular hybrid nanostructures formed through the electrostatic interaction of cationic chitosan polysaccharide molecules and end-functionalized PNIPAM chains bearing a negative carboxylate ion. Different combinations of long and short chains in respect to both macromolecular components at varying mixing ratios were investigated and stable complexes were formed in all cases. Their overall physicochemical properties regarding their mass, size, charge, structure, and morphology were thoroughly examined by means of light scattering, potentiometry, and fluorescence spectroscopy, as well as STEM and AFM imaging. In general, it was found that the individual characteristics of the formed complexes are directly correlated to the length of the chitosan molecules and the PNIPAM chains (long/short), the specific combination of the different components’ lengths, and their mixing ratio. Along these lines, the influence of the length of the chitosan molecule was proven particularly significant since it led to complexes of different component arrangements and internal/overall structures, i.e., either loose or dense. Due to the thermoresponsive nature of the PNIPAM chains, the various Chi/PNIPAM complexes had a similar temperature-dependent behavior, demonstrating fully reversible conformational changes upon the increase in temperature to above 35 °C. Moreover, they were also responsive to the increase in the ionic strength of the solution, exhibiting either shrinking or partial disassembly (depending on their initial conformation) as a result of the occurring charge screening. Finally, the potential use of these nanostructures as drug delivery vehicles was verified by the successful encapsulation of curcumin, owing to the hydrophobic interactions with the corresponding hydrophobic regions of the complexes. On the whole, the sum of the obtained results demonstrates the importance of the delicate balance between electrostatic and hydrophobic interactions when it comes to such structurally complex systems.

## Figures and Tables

**Table 1:** The sample codes, along with the corresponding volume, and the calculated molar and [+]/[−] charge ratios for the various Chi/PNIPAM complexes.

Sample Code *	Chi/PNIPAM Volume Ratio	PNIPAM/Chi Molar Ratio		[+]/[−] Charge Ratio	
		L1	S1	L1	S1
CompL1 or S1_4/1	4/1	2	0.02	410	424
CompL1 or S1_4/2	4/2	4	0.04	205	212
CompL1 or S1_4/4	4/4	8	0.08	102.5	106
		**L2**	**S2**	**L2**	**S2**
CompL1 or S2_4/1	4/1	13	0.11	68	70
CompL1 or S2_4/2	4/2	26	0.22	34	35
CompL1 or S2_4/4	4/4	52	0.44	17	17.5

* Where L and S stand for Chi162K and 1.5 K, and 1 and 2 stand for PNIPAM19K and 3 K, respectively.

**Table 2:** Charge density values for the various Chi/PNIPAM complexes.

Charge Density (meq/g)			
Sample	4/1	4/2	4/4
CompL1_	2.48	2.20	1.60
CompS1_	0.48	0.44	0.36
CompL2_	2.17	1.94	1.37
CompS2_	0.36	0.30	0.20

**Table 3:** Pyrene fluorescence *I*_1_/*I*_3_ intensity ratio values for the various Chi/PNIPAM complexes, calculated from the corresponding fluorescence spectra measured both at 25 and 45 °C.

*I*_1_/*I*_3_ Intensity Ratio						
	At 25 °C			At 45 °C		
Sample:	4/1	4/2	4/4	4/1	4/2	4/4
CompL1_	1.62	1.59	1.62	1.42	1.38	1.31
CompS1_	1.24	1.19	1.17	1.16	1.11	1.12
CompL2_	1.68	1.63	1.54	1.59	1.63	1.58
CompS2_	1.57	1.53	1.56	1.48	1.57	1.44

**Table 4:** The sample codes, the concentration of added and encapsulated CUR, along with the encapsulation efficiency EE%, for the CUR-loaded CompL1_4/4 samples.

Sample Code	Added *C*_CUR_ (μg/mL)	Encapsulated *C*_CUR_ (μg/mL)	EE%
CompL1_4/4 + 2.5%CUR	19.6	8.5	43.4
CompL1_4/4 + 5%CUR	38.5	17.9	46.5
CompL1_4/4 + 10%CUR	74.1	45.4	61.3

## Data Availability

Data are contained within the article and Supplementary Materials.

## Supplementary Materials

The following supporting information can be downloaded at https://www.mdpi.com/article/10.3390/nano15050350/s1: Table S1: Characteristics of the drug-loaded Chi/PNIPAM complexes; Figure S1: Preparation procedure for the drug-loaded Chi/PNIPAM complexes; Figure S2: UV–Vis calibration curve for CUR in ethanol; Table S2: Polyelectrolyte titration calculations for the various Chi/PNIPAM complexes; Figure S3: Polydispersity index for the various Chi/PNIPAM complexes; Figure S4: Pyrene fluorescence spectra for the various Chi/PNIPAM complexes; Figure S5: STEM images for some of the Chi/PNIPAM samples, showing larger clustered structures; Figures S6–S9: AFM images and the corresponding size measurements for the various Chi/PNIPAM complexes; Figures S10–S14: Temperature effect for the rest of the Chi/PNIPAM complexes, and the two PNIPAM samples; Figures S15–S18: Ionic strength effect for the rest of the Chi/PNIPAM complexes, the two PNIPAM, and the two chitosan samples.

## References

[1] Vargason A.M., Anselmo A.C., Mitragotri S. (2021). The Evolution of Commercial Drug Delivery Technologies. Nat. Biomed. Eng..

[2] Wu D., Zhu L., Li Y., Zhang X., Xu S., Yang G., Delair T. (2020). Chitosan-Based Colloidal Polyelectrolyte Complexes for Drug Delivery: A Review. Carbohydr. Polym..

[3] Iqbal Y., Ahmed I., Irfan M.F., Chatha S.A.S., Zubair M., Ullah A. (2023). Recent Advances in Chitosan-Based Materials; The Synthesis, Modifications and Biomedical Applications. Carbohydr. Polym..

[4] Pramanik S., Sali V. (2021). Connecting the Dots in Drug Delivery: A Tour d’horizon of Chitosan-Based Nanocarriers System. Int. J. Biol. Macromol..

[5] Iacob A.T., Lupascu F.G., Apotrosoaei M., Vasincu I.M., Tauser R.G., Lupascu D., Giusca S.E., Caruntu I.-D., Profire L. (2021). Recent Biomedical Approaches for Chitosan Based Materials as Drug Delivery Nanocarriers. Pharmaceutics.

[6] Abourehab M.A.S., Pramanik S., Abdelgawad M.A., Abualsoud B.M., Kadi A., Ansari M.J., Deepak A. (2022). Recent Advances of Chitosan Formulations in Biomedical Applications. Int. J. Mol. Sci..

[7] Bashir S.M., Ahmed Rather G., Patrício A., Haq Z., Sheikh A.A., Shah M.Z.U.H., Singh H., Khan A.A., Imtiyaz S., Ahmad S.B. (2022). Chitosan Nanoparticles: A Versatile Platform for Biomedical Applications. Materials.

[8] Gao Y., Wu Y. (2022). Recent Advances of Chitosan-Based Nanoparticles for Biomedical and Biotechnological Applications. Int. J. Biol. Macromol..

[9] Jha R., Mayanovic R.A. (2023). A Review of the Preparation, Characterization, and Applications of Chitosan Nanoparticles in Nanomedicine. Nanomaterials.

[10] Haider A., Khan S., Iqbal D.N., Shrahili M., Haider S., Mohammad K., Mohammad A., Rizwan M., Kanwal Q., Mustafa G. (2024). Advances in Chitosan-Based Drug Delivery Systems: A Comprehensive Review for Therapeutic Applications. Eur. Polym. J..

[11] Lingait D., Rahagude R., Gaharwar S.S., Das R.S., Verma M.G., Srivastava N., Kumar A., Mandavgane S. (2024). A Review on Versatile Applications of Biomaterial/Polycationic Chitosan: An Insight into the Structure-Property Relationship. Int. J. Biol. Macromol..

[12] Desai N., Rana D., Salave S., Gupta R., Patel P., Karunakaran B., Sharma A., Giri J., Benival D., Kommineni N. (2023). Chitosan: A Potential Biopolymer in Drug Delivery and Biomedical Applications. Pharmaceutics.

[13] Guo Y., Qiao D., Zhao S., Liu P., Xie F., Zhang B. (2024). Biofunctional Chitosan–Biopolymer Composites for Biomedical Applications. Mater. Sci. Eng. R Rep..

[14] Drăgan E.S., Mihai M., Schwarz S. (2009). Complex nanoparticles based on chitosan and ionic/nonionic strong polyanions: Formation, stability, and application. ACS Appl. Mater. Interfaces.

[15] Mihai M., Ghiorghiţă C.A., Stoica I., Niţă L., Popescu I., Fundueanu G. (2011). New polyelectrolyte complex particles as colloidal dispersions based on weak synthetic and/or natural polyelectrolytes. Express Polym. Lett..

[16] Nazarova A., Yakimova L., Mostovaya O., Kulikova T., Mikhailova O., Evtugyn G., Ganeeva I., Bulatov E., Stoikov I. (2022). Encapsulation of the Quercetin with Interpolyelectrolyte Complex Based on Pillar[5]Arenes. J. Mol. Liq..

[17] Gileva A., Trushina D., Yagolovich A., Gasparian M., Kurbanova L., Smirnov I., Burov S., Markvicheva E. (2023). Doxorubicin-Loaded Polyelectrolyte Multilayer Capsules Modified with Antitumor DR5-Specific TRAIL Variant for Targeted Drug Delivery to Tumor Cells. Nanomaterials.

[18] Zhang Y., Guo D., Shen X., Tang Z., Lin B. (2024). Recoverable and Degradable Carboxymethyl Chitosan Polyelectrolyte Hydrogel Film for Ultra Stable Encapsulation of Curcumin. Int. J. Biol. Macromol..

[19] Lanzalaco S., Armelin E. (2017). Poly(*N*-Isopropylacrylamide) and Copolymers: A Review on Recent Progresses in Biomedical Applications. Gels.

[20] Bao H., Ping Y., Pan Y., Li L., Li J., Gan L.H. (2012). Thermo-Responsive Transfection of DNA Complexes with Well-Defined Chitosan Terpolymers. Soft Matter.

[21] Qi M., Li G., Yu N., Meng Y., Liu X. (2014). Synthesis of Thermo-Sensitive Polyelectrolyte Complex Nanoparticles from CS-g-PNIPAM and SA-g-PNIPAM for Controlled Drug Release. Macromol. Res..

[22] Pitakchatwong C., Chirachanchai S. (2017). Thermo-Magnetoresponsive Dual Function Nanoparticles: An Approach for Magnetic Entrapable–Releasable Chitosan. ACS Appl. Mater. Interfaces.

[23] Ziminska M., Wilson J.J., McErlean E., Dunne N., McCarthy H.O. (2020). Synthesis and Evaluation of a Thermoresponsive Degradable Chitosan-Grafted PNIPAAm Hydrogel as a “Smart” Gene Delivery System. Materials.

[24] Zaharia M.-M., Bucatariu F., Karayianni M., Lotos E.-D., Mihai M., Pispas S. (2024). Synthesis of Thermoresponsive Chitosan-Graft-Poly(*N*-Isopropylacrylamide) Hybrid Copolymer and Its Complexation with DNA. Polymers.

[25] Ma P.L., Lavertu M., Winnik F.M., Buschmann M.D. (2009). New Insights into Chitosan−DNA Interactions Using Isothermal Titration Microcalorimetry. Biomacromolecules.

[26] Giaouzi D., Pispas S. (2019). Synthesis and Self-assembly of Thermoresponsive Poly(*N*-isopropylacrylamide)-b-poly(Oligo Ethylene Glycol Methyl Ether Acrylate) Double Hydrophilic Block Copolymers. J. Polym. Sci. Part A Polym. Chem..

[27] Winnik F.M. (1993). Photophysics of Preassociated Pyrenes in Aqueous Polymer Solutions and in Other Organized Media. Chem. Rev..

[28] Hugerth A.M. (2001). Micropolarity and Microviscosity of Amitriptyline and Dextran Sulfate/Carrageenan-Amitriptyline Systems: The Nature of Polyelectrolyte–Drug Complexes. J. Pharm. Sci..

[29] Piñeiro L., Novo M., Al-Soufi W. (2015). Fluorescence Emission of Pyrene in Surfactant Solutions. Adv. Colloid Interface Sci..

[30] Rinaudo M. (2017). Physicochemical behaviour of semi-rigid biopolymers in aqueous medium. Food Hydrocoll..

[31] Zhang Y., Furyk S., Bergbreiter D.E., Cremer P.S. (2005). Specific Ion Effects on the Water Solubility of Macromolecules: PNIPAM and the Hofmeister Series. J. Am. Chem. Soc..

[32] Humphreys B.A., Wanless E.J., Webber G.B. (2018). Effect of Ionic Strength and Salt Identity on Poly(N-Isopropylacrylamide) Brush Modified Colloidal Silica Particles. J. Colloid Interface Sci..

[33] Pan Y., Suo Z., Lu T. (2022). A Thermodynamic Model of Phase Transition of Poly(N-Isopropylacrylamide) Hydrogels in Ionic Solutions. Int. J. Solids Struct..

[34] Hsu K.Y., Ho C.T., Pan M.H. (2023). The Therapeutic Potential of Curcumin and Its Related Substances in Turmeric: From Raw Material Selection to Application Strategies. J. Food Drug Anal..

[35] Govindaraju S., Rengaraj A., Arivazhagan R., Huh Y.S., Yun K. (2018). Curcumin-Conjugated Gold Clusters for Bioimaging and Anticancer Applications. Bioconjug. Chem..

[36] Liu M., Teng C.P., Win K.Y., Chen Y., Zhang X., Yang D.P., Li Z., Ye E. (2019). Polymeric Encapsulation of Turmeric Extract for Bioimaging and Antimicrobial Applications. Macromol. Rapid Commun..

[37] Selianitis D., Forys A., Trzebicka B., Alemayehu A., Tyrpekl V., Pispas S. (2022). Amphiphilic P(OEGMA-Co-DIPAEMA) Hyperbranched Copolymer/Magnetic Nanoparticle Hybrid Nanostructures by Co-Assembly. Nanomanufacturing.

[38] Skandalis A., Selianitis D., Sory D.R., Rankin S.M., Jones J.R., Pispas S. (2022). Poly(2-(Dimethylamino) Ethyl Methacrylate)-b-Poly(Lauryl Methacrylate)-b-Poly(Oligo Ethylene Glycol Methacrylate) Triblock Terpolymer Micelles as Drug Delivery Carriers for Curcumin. J. Appl. Polym. Sci..

