# Supplementary material for: Natural–Synthetic Hybrid Nanostructures Formed Through the Interaction of Chitosan with Carboxylate-Ended PNIPAM: Structure and Curcumin Encapsulation
Source: Nanomaterials (Basel). 2025 Feb 24;15(5):350. doi: 10.3390/nano15050350 (PMC11901671; doi:10.3390/nano15050350)
Supplement: Supplementary file 1 [file nanomaterials-15-00350-s001.zip › nanomaterials-3480768-supplementary.pdf]

Supplementary Materials

Natural–Synthetic Hybrid Nanostructures Formed through the Interaction of Chitosan with Carboxylate Ended PNIPAM: Structure and Curcumin Encapsulation

Elena-Daniela Lotos [1], Maria Karayianni [1], Ana-Lavinia Vasiliu [1], Marcela Mihai [1,*] and Stergios Pispas [1,2,*]

[1] Petru Poni Institute of Macromolecular Chemistry, 41A Grigore Ghica Voda Alley, 700487 Iasi, Romania; daniela.lotos@icmpp.ro (E.-D.L.); m.karayianni@icmpp.ro (M.K.); vasiliu.lavinia@icmpp.ro (A.-L.V.)
[2] Theoretical and Physical Chemistry Institute, National Hellenic Research Foundation, 48 Vassileos Constantinou Ave., 116 35 Athens, Greece
* Correspondence: marcela.mihai@icmpp.ro (M.M.); pispas@eie.gr (S.P.)

Drug Loading Procedure for the Chi/PNIPAM Complexes

The drug loading capacity of the CompL1_4/4 sample was investigated using curcumin (CUR) as a model hydrophobic drug and the experimental procedure is described in detail in Section 2.3. Table S1 summarizes the sample codes, corresponding masses of the constituents, the drug concentration and the total volume for each solution. The incorporation of CUR in the solution of the Chi162K/PNIPAM19K complex was performed at 40 °C utilizing a water bath as seen in Figure S1a, while the final stable CUR-loaded solutions/dispersions for the three different nominal loading concentrations examined are shown in Figure S1b. For the determination of the actual concentration of CUR in the final solutions a calibration curve of CUR in ethanol was constructed by measuring the absorbance at 427 nm for several calibration solutions corresponding to the concentration range of 0.5 to 5 μg/mL, as shown in Figure S2.

Table S1. The sample codes, the mass of Chi162K, PNIPAM19K and CUR in the solution, the nominal concentration of CUR both as % *w*/*w* in regard to the mass of the initial complex and in μg/mL, along with the total volume of each solution, for the CUR loaded Chi162K/PNIPAM19K complexes.

Sample Code	$m_{Chi162K}$ (mg)	$m_{PNIPAM19K}$ (mg)	m_{CUR} (mg)	C_{CUR} (% *w*/*w*)	C_{CUR} (μg/mL)	Total volume (mL)
CompL1_4/4	8	8	0	0	0	20
CompL1_4/4+2.5%CUR			0.1	2.5	19.6	5.1
CompL1_4/4+5%CUR	2	2	0.2	5	38.5	5.2
CompL1_4/4+10%CUR			0.4	10	74.1	5.4

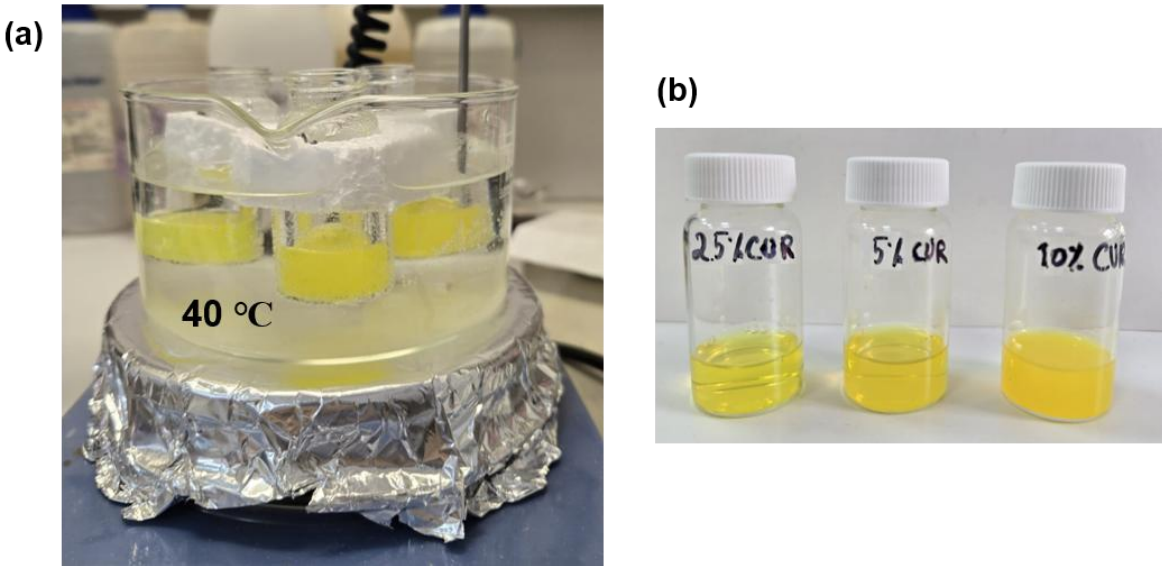

Figure S1. (**a**) Preparation procedure and (**b**) obtained solutions/dispersions for the three different CUR loaded Chi162K+PNIPAM19K complexes.

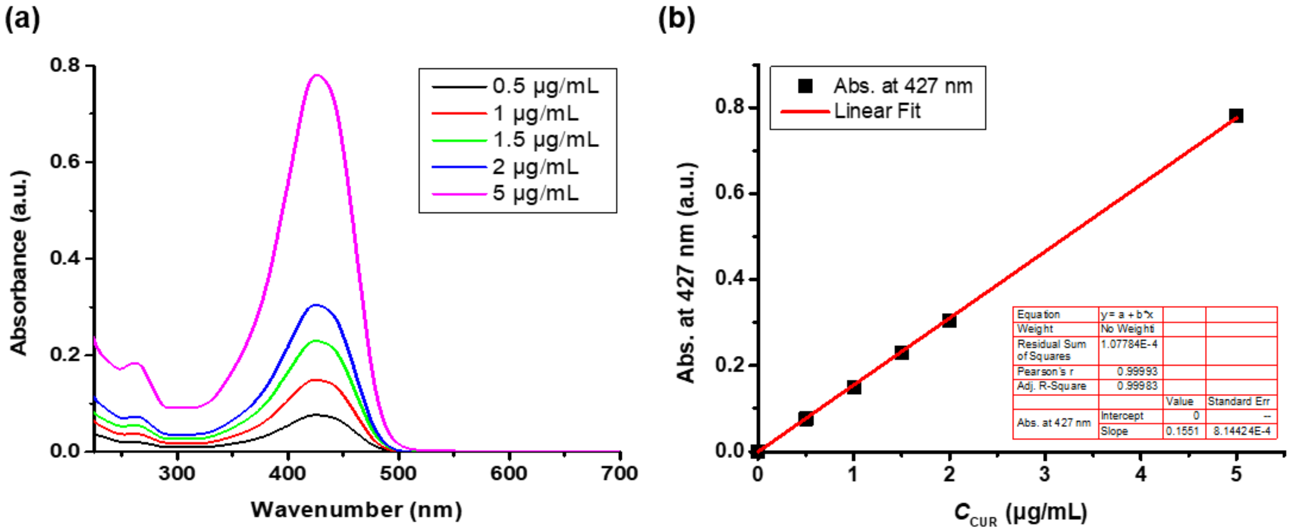

Figure S2. (**a**) UV–Vis spectra for CUR in EtOH at various concentrations, along with (**b**) the corresponding calibration curve.

Polydispersity Index for the Chi/PNIPAM Complexes

The polydispersity index values obtained from the DLS measurements of the various Chi/PNIPAM complexes, are presented in Figure S23. All four systems exhibit rather high polydispersity values since different scattering populations coexist in solution, as evidenced by the various peaks discerned in the corresponding size distributions shown in Figures 1 and 2 of the main manuscript. Upon comparison of the different systems, it is obvious that the complexes formed with the short chitosan (CompS1 and S2) have a higher polydispersity index, in the range of 0.8–1, than the ones formed with the long one (CompL1 and L2), which show values about 0.5 to 0.6. This is a direct consequence of the fact that for both CompS1 and S2 series a greater difference in the size of the various populations is observed, mainly due to the significantly larger size of Peak 3. Accordingly, a slightly higher polydispersity is seen in the case of CompL2 series in comparison to the ones for CompL1, as also evidenced by the somewhat broader peaks, indicating a larger heterogeneity of sizes for each population. Finaly, the CompS2 series exhibits quite lower polydispersity than CompS1, since two peaks instead of three are distinguished.

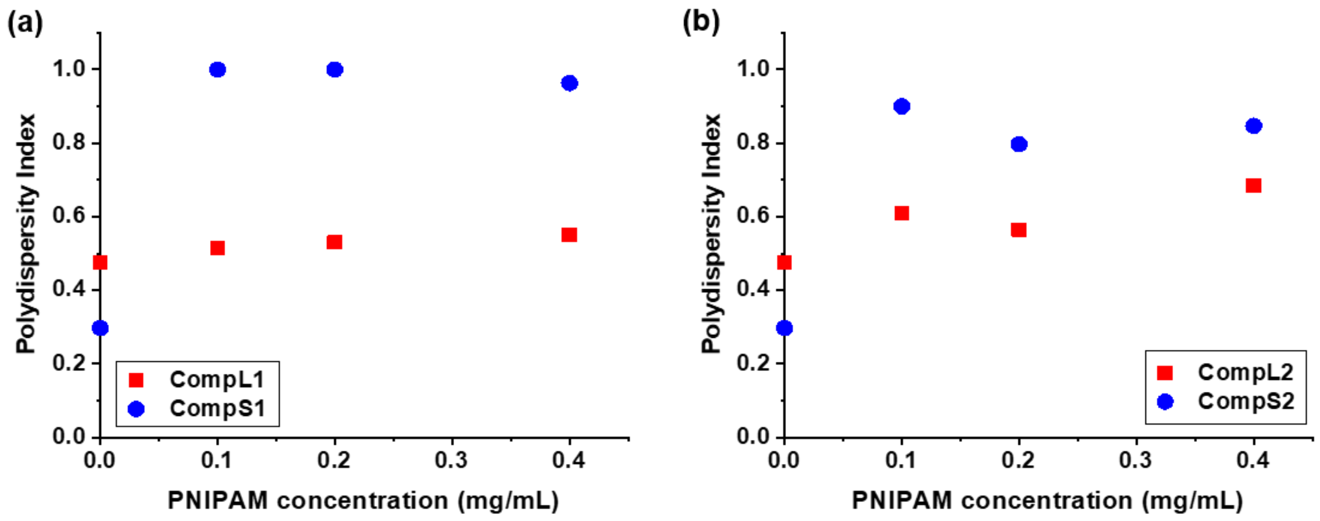

Figure S3. Polydispersity index for the (**a**) CompL1 and CompS1 or (**b**) CompL2 and CompS2 series of complexes.

Polyelectrolyte Titration for the Chi/PNIPAM Complexes

For the determination of the charge density of the various Chi/PNIPAM complexes, 1 mL from each sample was titrated with a strong polyelectrolyte solution (i.e., PESNa with a 0.874 mM concentration) as described in Section 2.5 of the main manuscript. From the total volume of PESNa solution needed for the full titration of the charges of the complexes, the total amount of polyelectrolyte moles and thus the milliequivalent charges (meq) are derived. Subsequently, the charge density of the complexes is calculated according to the total mass of each sample and expressed as meq of charges per g of sample (meq/g). The corresponding values for the various Chi/PNIPAM complexes are summarized in Table S2.

Table S2. Polyelectrolyte titration calculations for the various Chi/PNIPAM complexes.

Sample Code	Polyelectrolyte total volume (mL)	Polyelectrolyte moles (10^{-3} mmol)	Complex total mass (mg)	Charge density (meq/g)
CompL1_4/1	1.421	1.242	0.5	2.484
CompL1_4/2	1.513	1.322	0.6	2.204
CompL1_4/4	1.466	1.281	0.8	1.602
CompS1_4/1	0.275	0.240	0.5	0.481
CompS1_4/2	0.301	0.263	0.6	0.438
CompS1_4/4	0.332	0.290	0.8	0.363
CompL2_4/1	1.239	1.083	0.5	2.166
CompL2_4/2	1.330	1.162	0.6	1.937
CompL2_4/4	1.258	1.099	0.8	1.374
CompS2_4/1	0.204	0.178	0.5	0.357
CompS2_4/2	0.203	0.177	0.6	0.296
CompS2_4/4	0.180	0.157	0.8	0.197

Pyrene Fluorescence Spectra for the Chi/PNIPAM Complexes

The fluorescence spectra obtained with the addition of pyrene (in a final concentration of 1 μM) to the various Chi/PNIPAM solutions/dispersions of complexes measured both at 25 and 45 °C are displayed in Figure S4. The corresponding I_1/I_3 intensity ratio values for each spectrum are also shown, as well as being listed in Table 3 of the main manuscript.

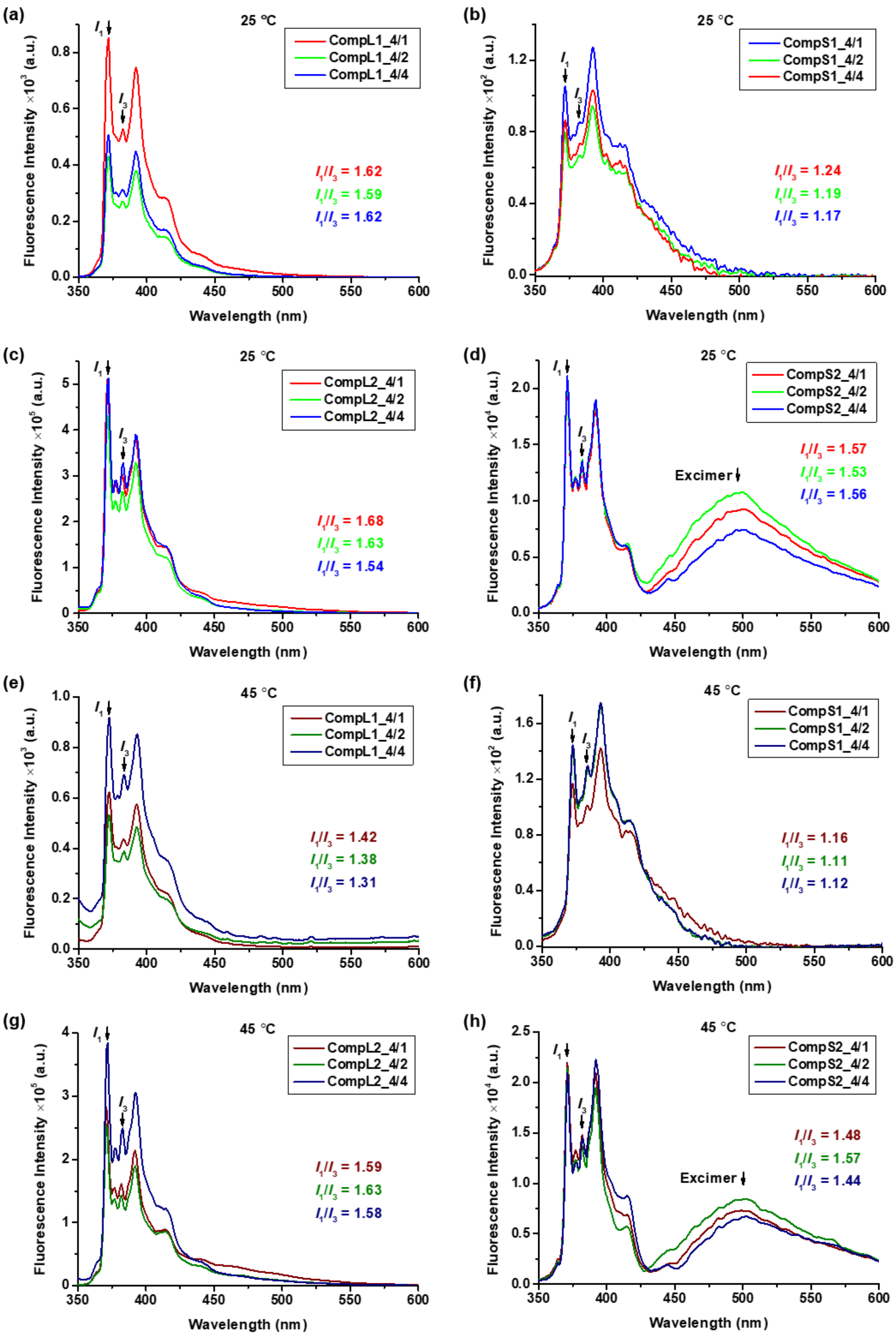

Figure S4. Fluorescence spectra with addition of pyrene (excitation at 335 nm), measured both at 25 °C (top two rows) and 45 °C (bottom two rows), for the (**a**,**e**) CompL1, (**b**,**f**) CompS1, (**c**,**g**) CompL2, and (**d**,**h**) CompS2 series of complexes. The corresponding I_1/I_3 intensity ratio values are also shown.

STEM Images for the Chi/PNIPAM Complexes

Additional STEM images for some of the Chi/PNIPAM samples showing representative larger structures, most probably clusters of primary complexes, are presented in Figure S5.

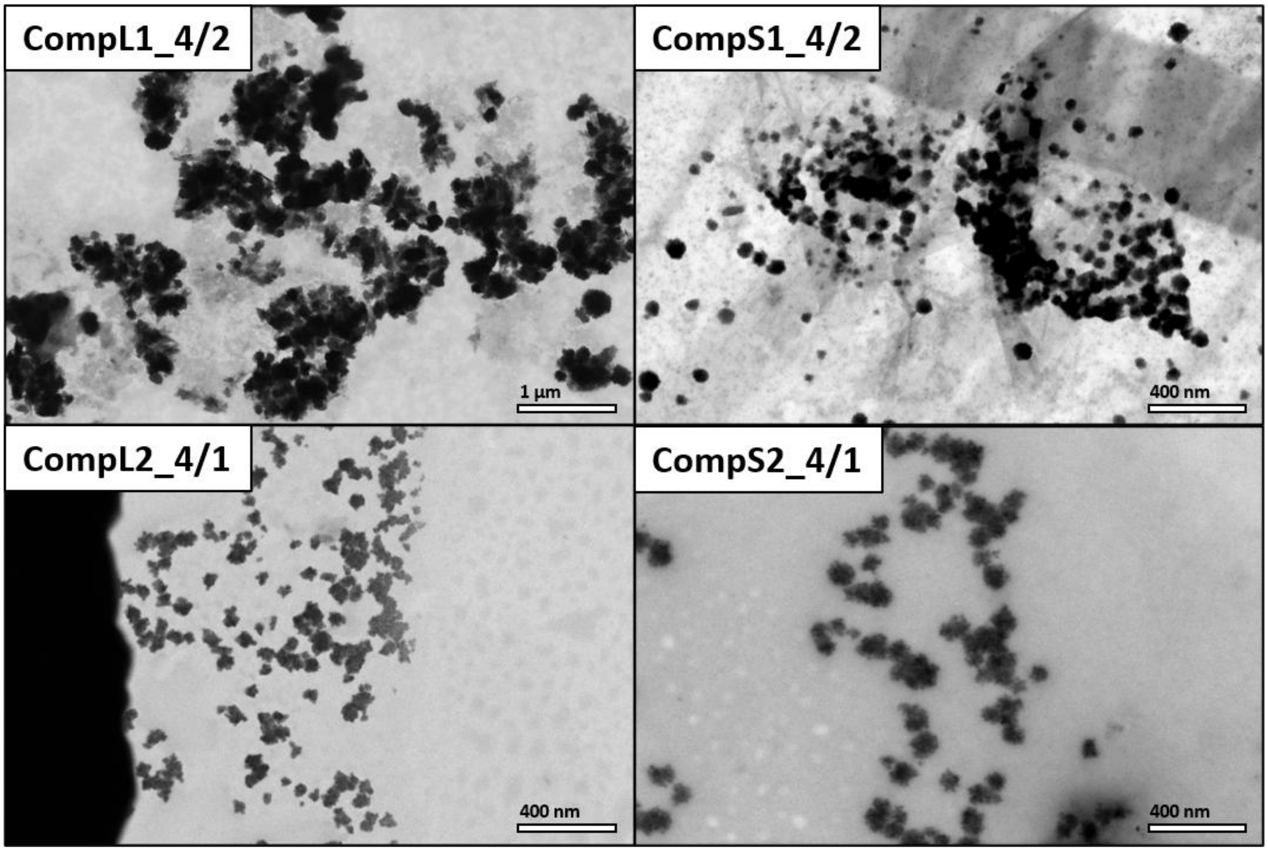

Figure S5. STEM images for some of the Chi/PNIPAM samples, showing larger structures.

AFM Images for the Chi/PNIPAM Complexes

The analysis of the AFM images obtained for all four series of the Chi/PNIPAM complexes, showing the sizes of the various individual particles that were visualized, is given in the following Figures S6–9.

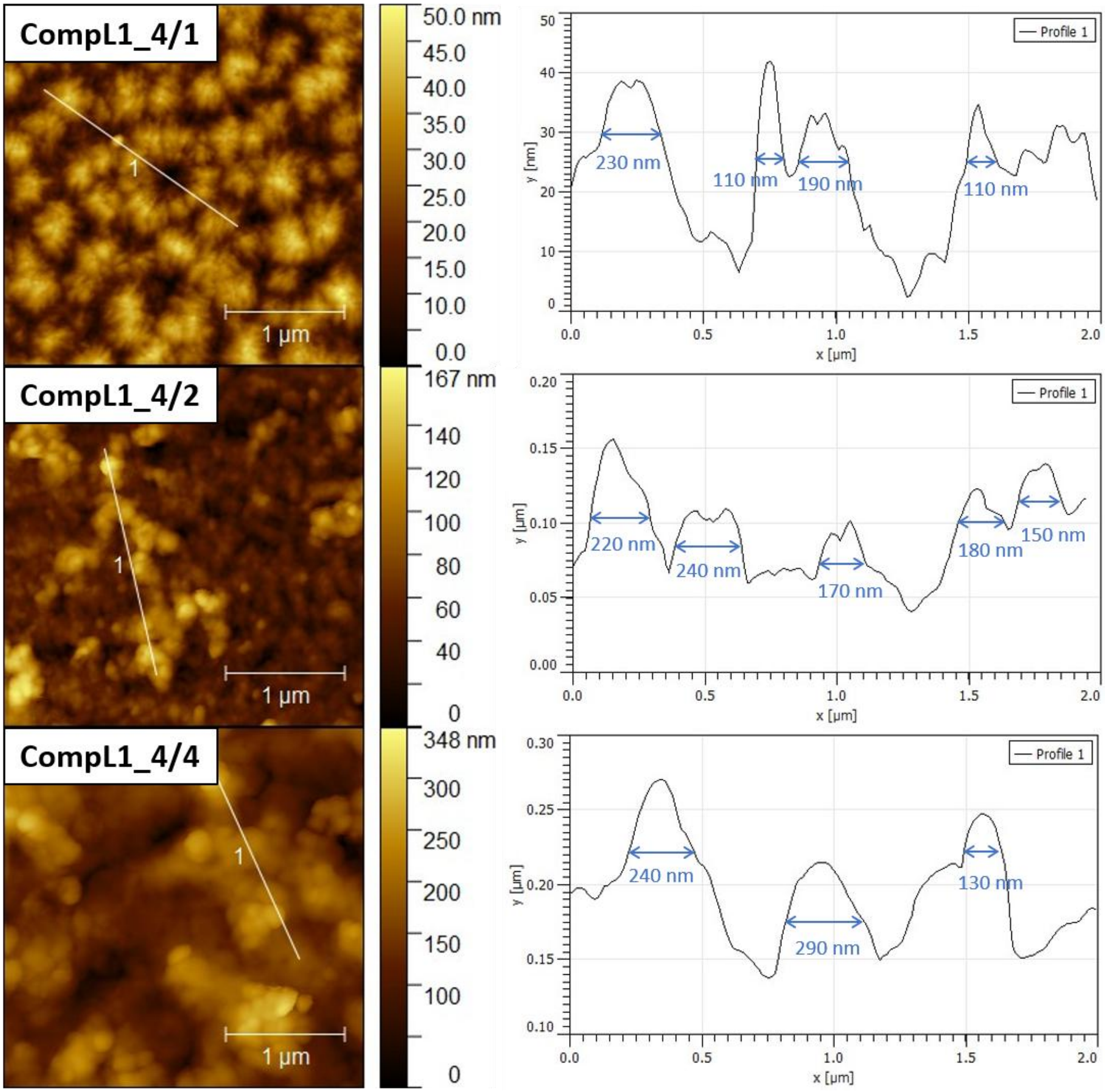

Figure S6. AFM images and the corresponding size measurements for the CompL1 series of samples. Profiles are taken along the lines marked as 1 in the images.

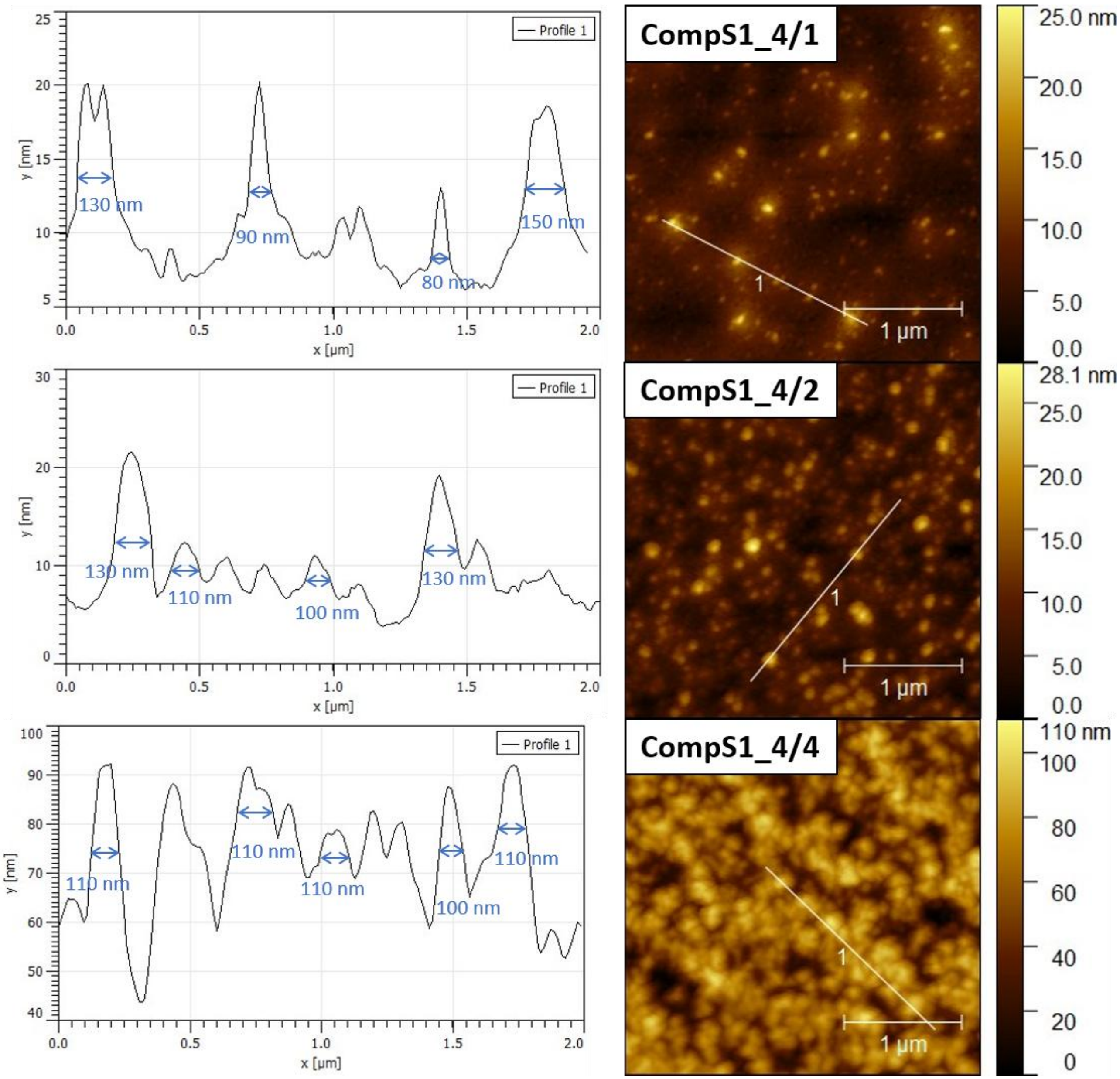

Figure S7. AFM images and the corresponding size measurements for the CompS1 series of samples. Profiles are taken along the lines marked as 1 in the images.

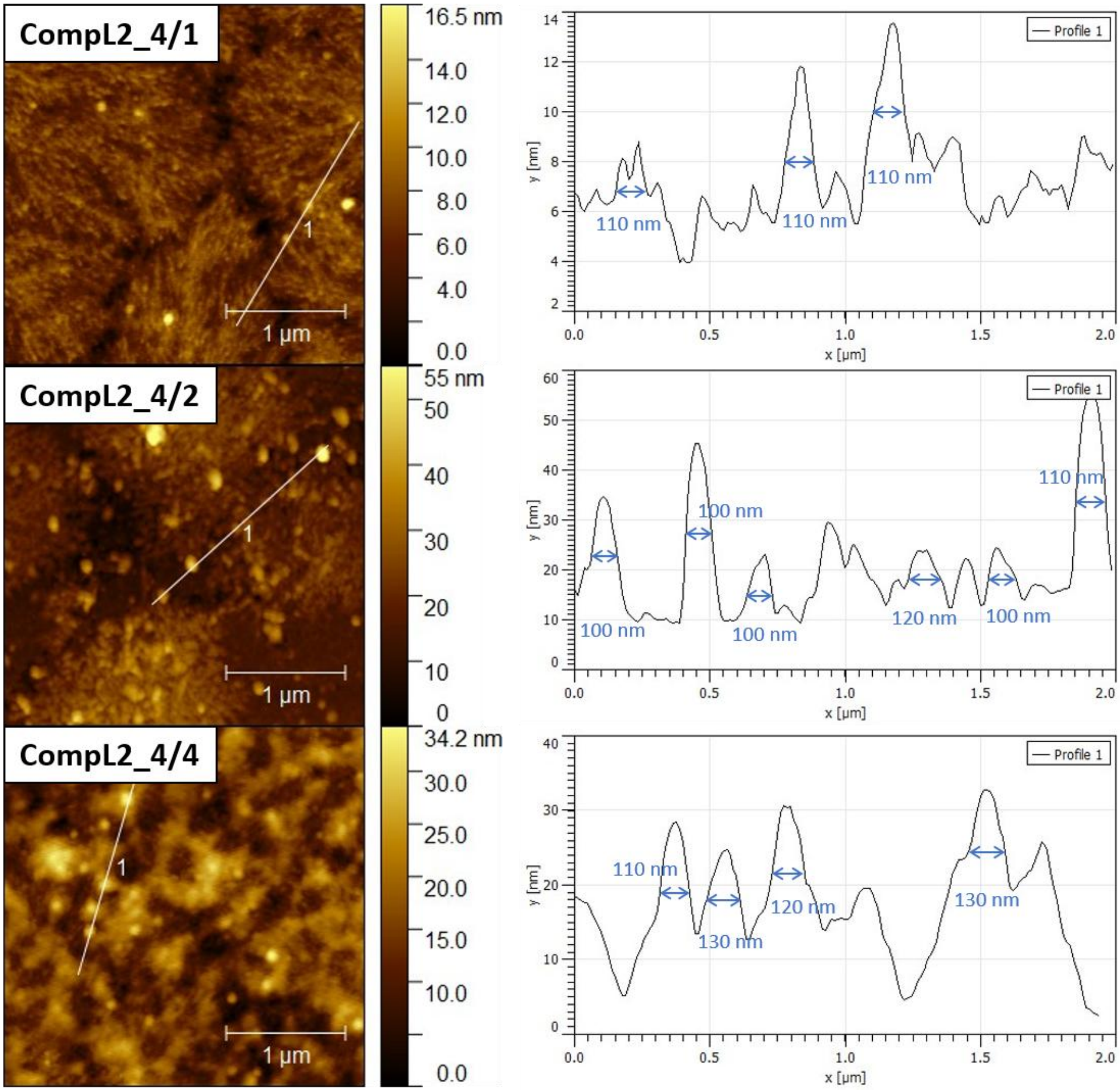

Figure S8. AFM images and the corresponding size measurements for the CompL2 series of samples. Profiles are taken along the lines marked as 1 in the images.

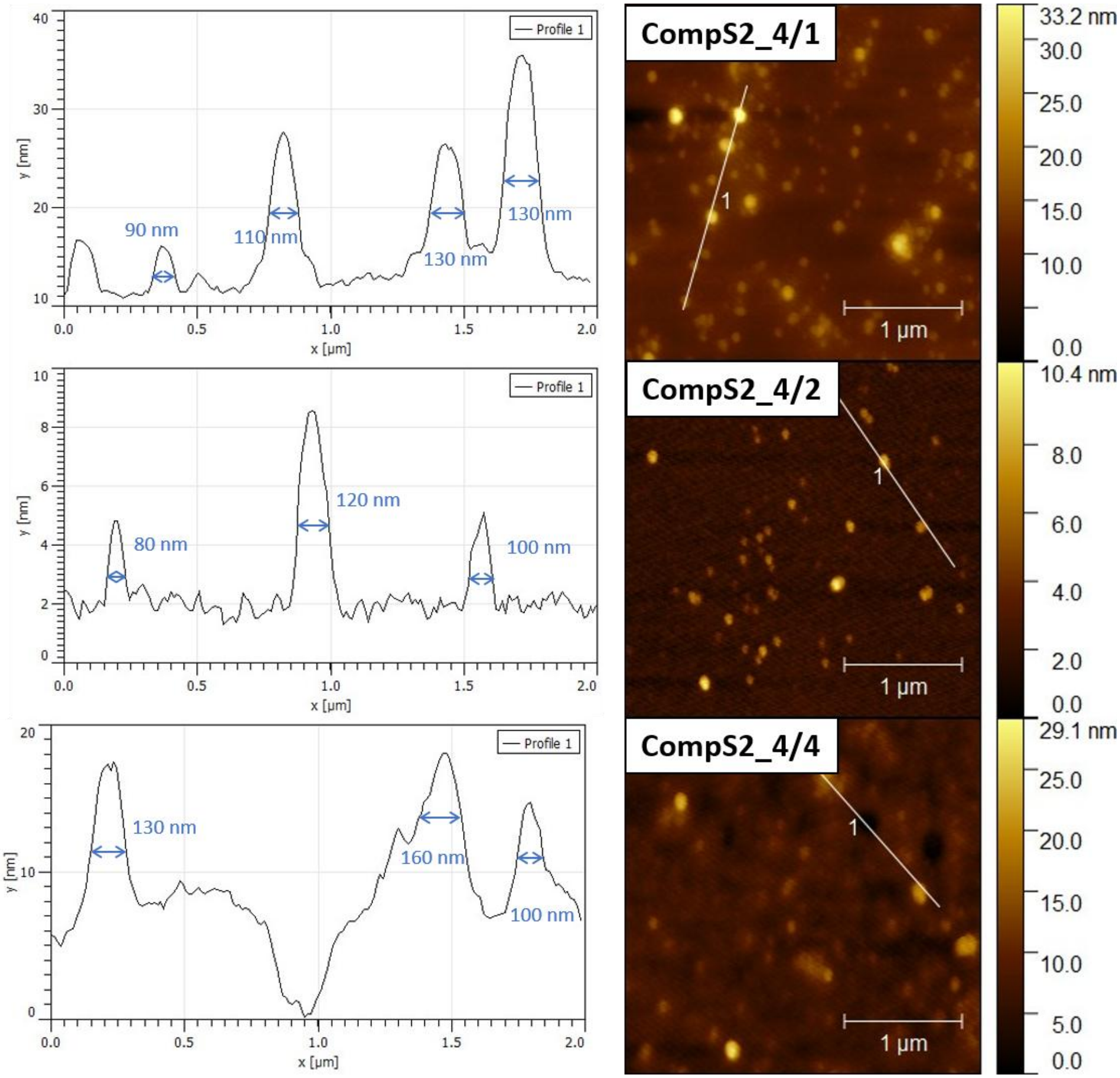

Figure S9. AFM images and the corresponding size measurements for the CompS2 series of samples. Profiles are taken along the lines marked as 1 in the images.

Temperature Response of the Chi/PNIPAM Complexes and the PNIPAM Samples

Additional temperature dependent DLS measurements were performed for the rest of the Chi/PNIPAM complexes, as well as for the two PNIPAM samples and the obtained results in regard to the scattered intensity, peaks' sizes and corresponding size distributions are shown in the following Figures S10–14. It should be noted that after the heating up to 45 °C the samples were brought back to 25 °C and measured again (so as to examine the reversibility of the system), with these measurements being marked as AH (i.e., after heating).

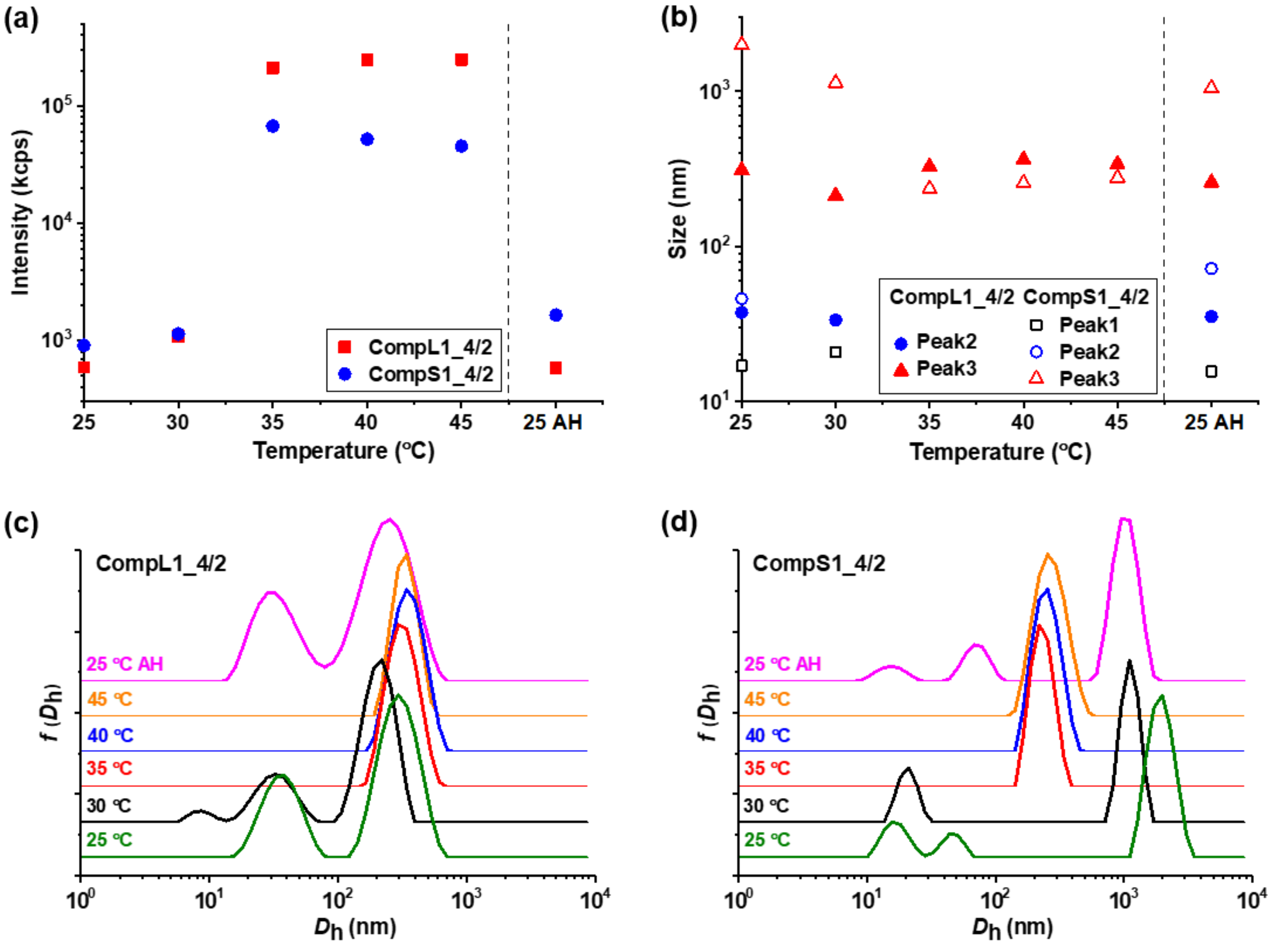

Figure S10. Temperature effect on the (**a**) scattered intensity, and (**b**) peaks' size, derived from the corresponding (**c**, **d**) size distributions, for the CompLorS1_4/2 samples.

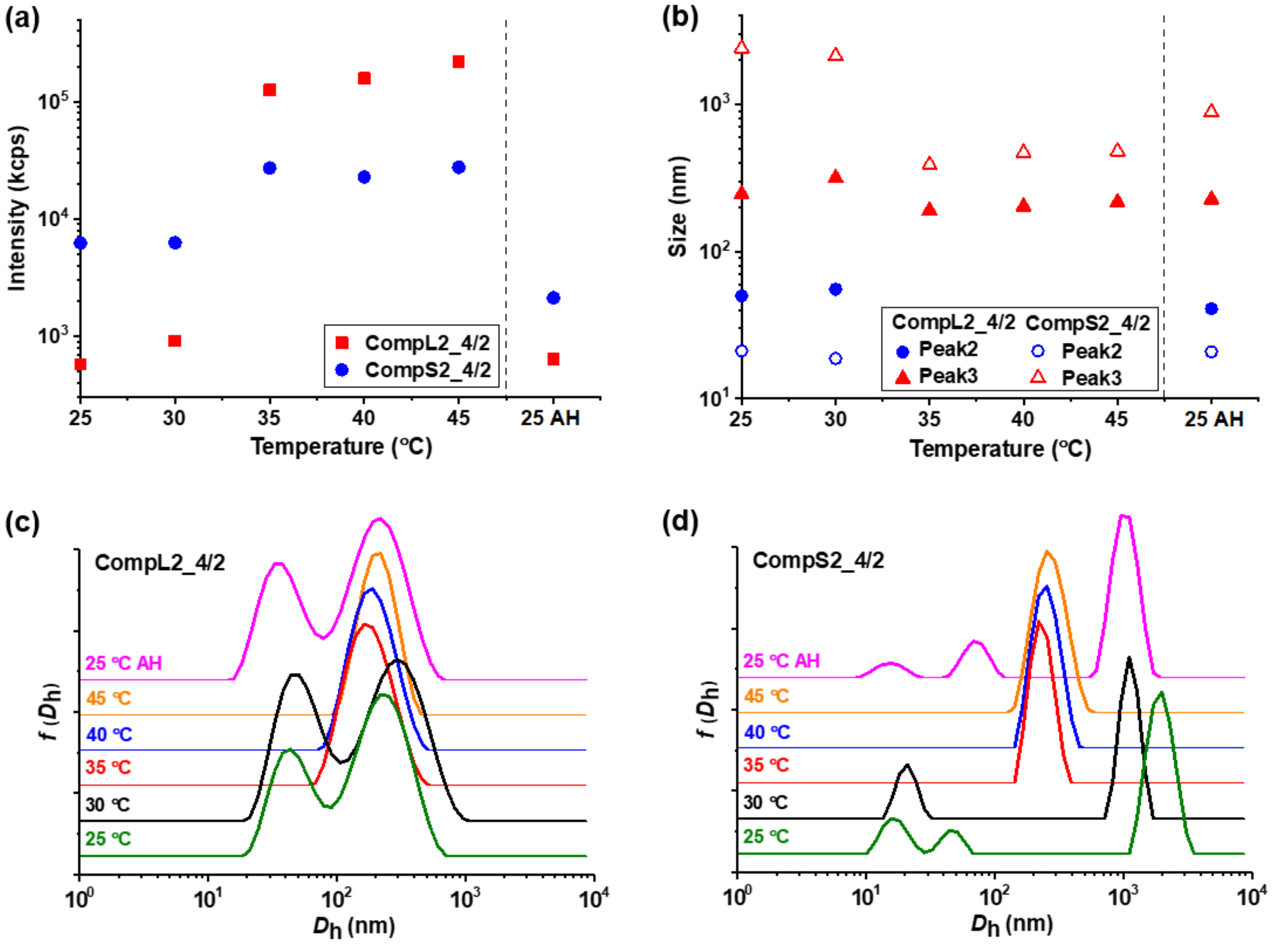

Figure S11. Temperature effect on the (**a**) scattered intensity, and (**b**) peaks' size, derived from the corresponding (**c**, **d**) size distributions, for the CompLorS2_4/2 samples.

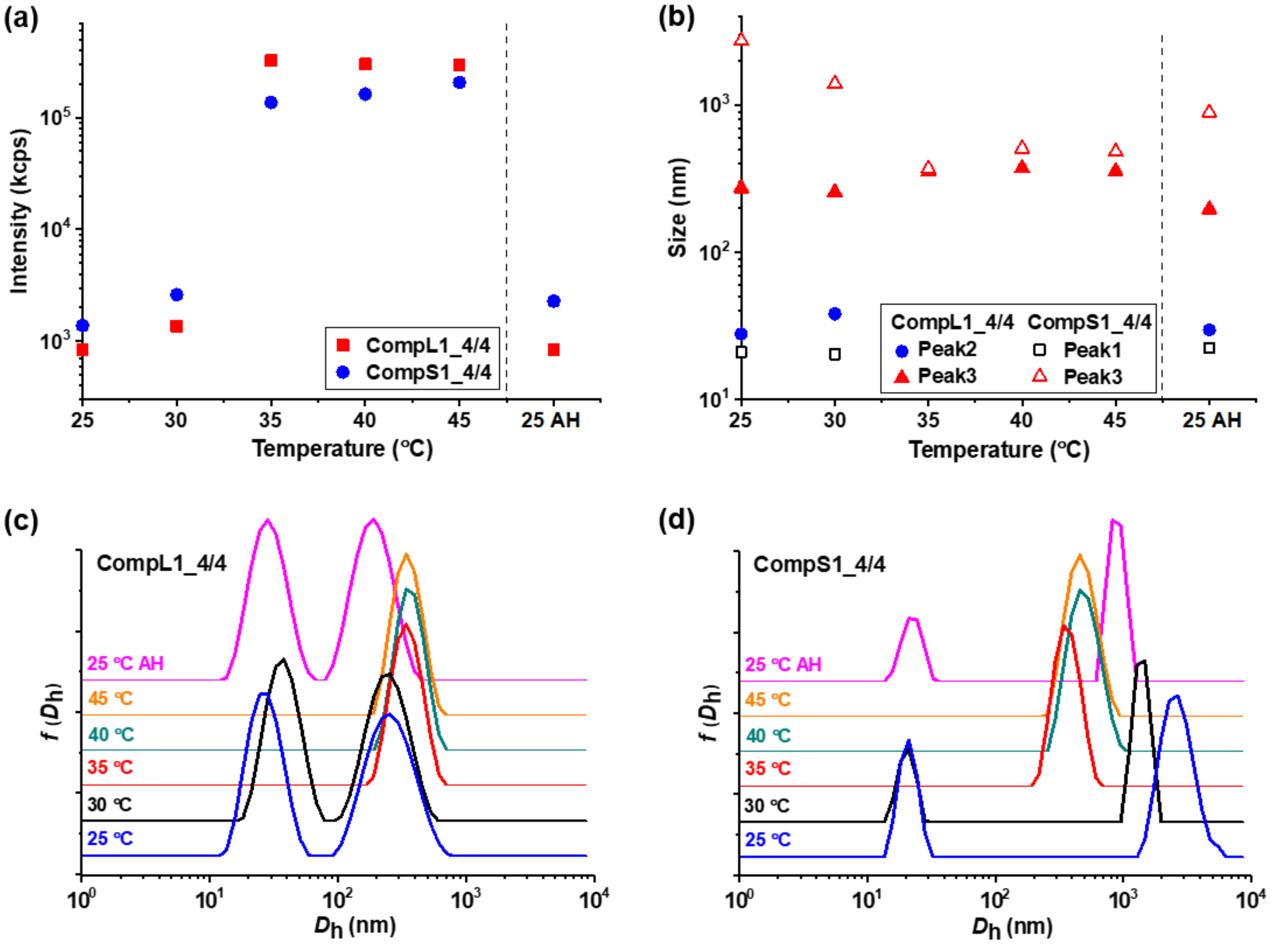

Figure S12. Temperature effect on the (**a**) scattered intensity, and (**b**) peaks' size, derived from the corresponding (**c**, **d**) size distributions, for the CompLorS1_4/4 samples.

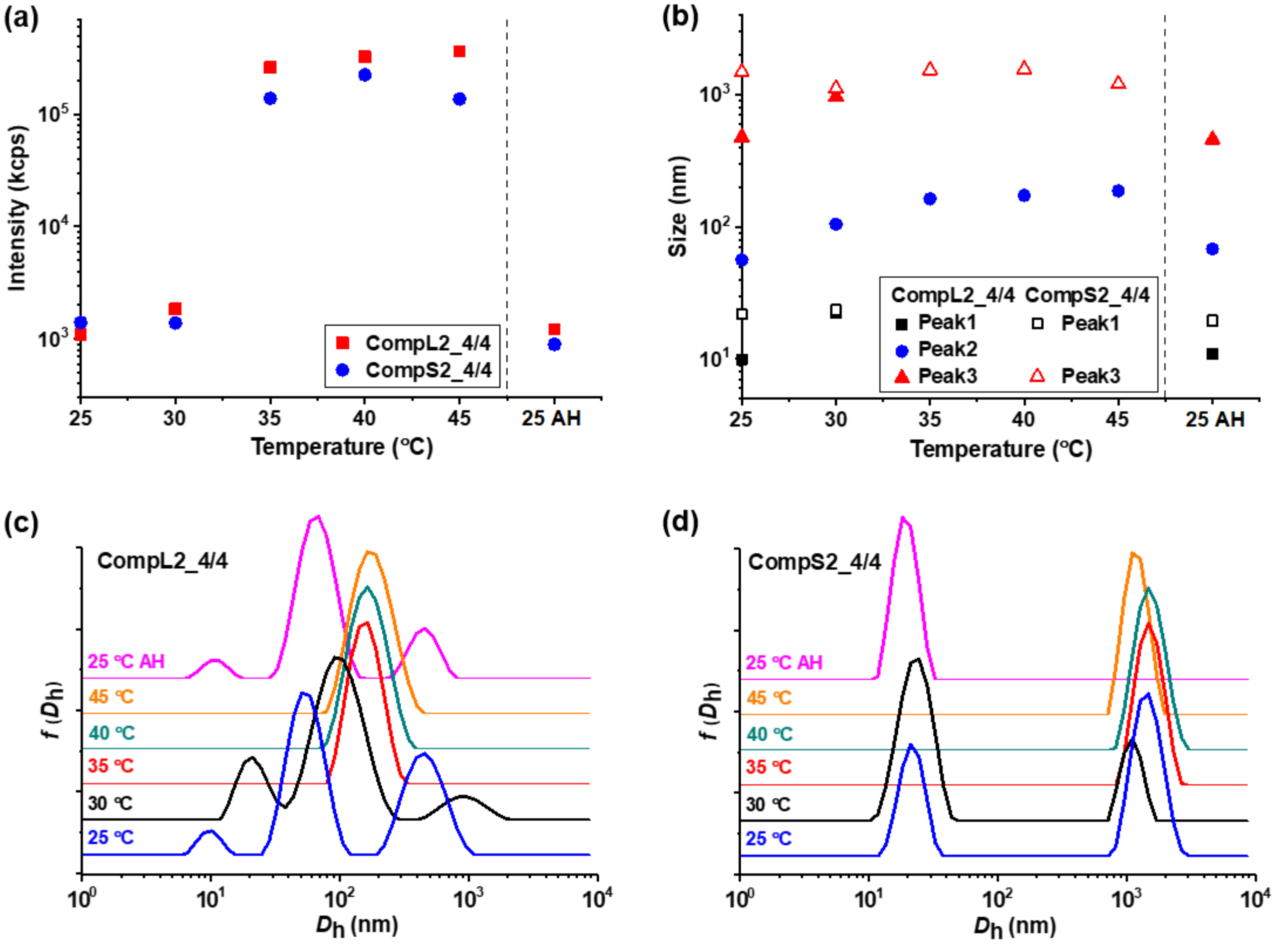

Figure S13. Temperature effect on the (**a**) scattered intensity, and (**b**) peaks' size, derived from the corresponding (**c**, **d**) size distributions, for the CompLorS2_4/4 samples.

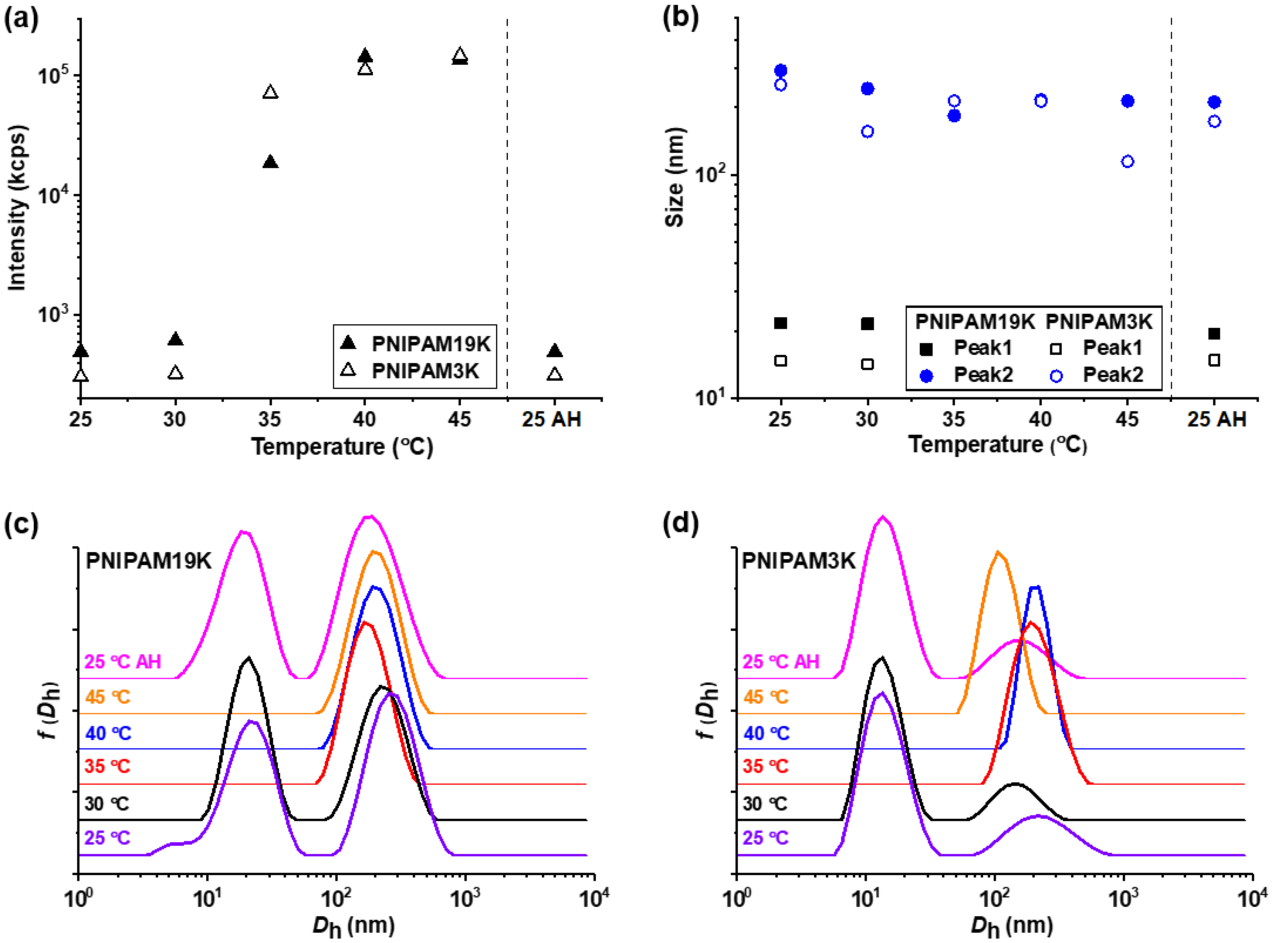

Figure S14. Temperature effect on the (**a**) scattered intensity, and (**b**) peaks' size, derived from the corresponding (**c**, **d**) size distributions, for the two PNIPAM samples.

Ionic Strength Effect on the Chi/PNIPAM Complexes and the PNIPAM and Chitosan Samples

The effects of ionic strength on the formed complexes were also investigated by DLS measurements for the 4/4 volume ratio samples of the four series of Chi/PNIPAM complexes, as well as the two PNIPAM and two chitosan samples utilized for complex formation. The obtained results regarding the scattered intensity, peaks' sizes and corresponding size distributions are shown in the following Figures S15–18.

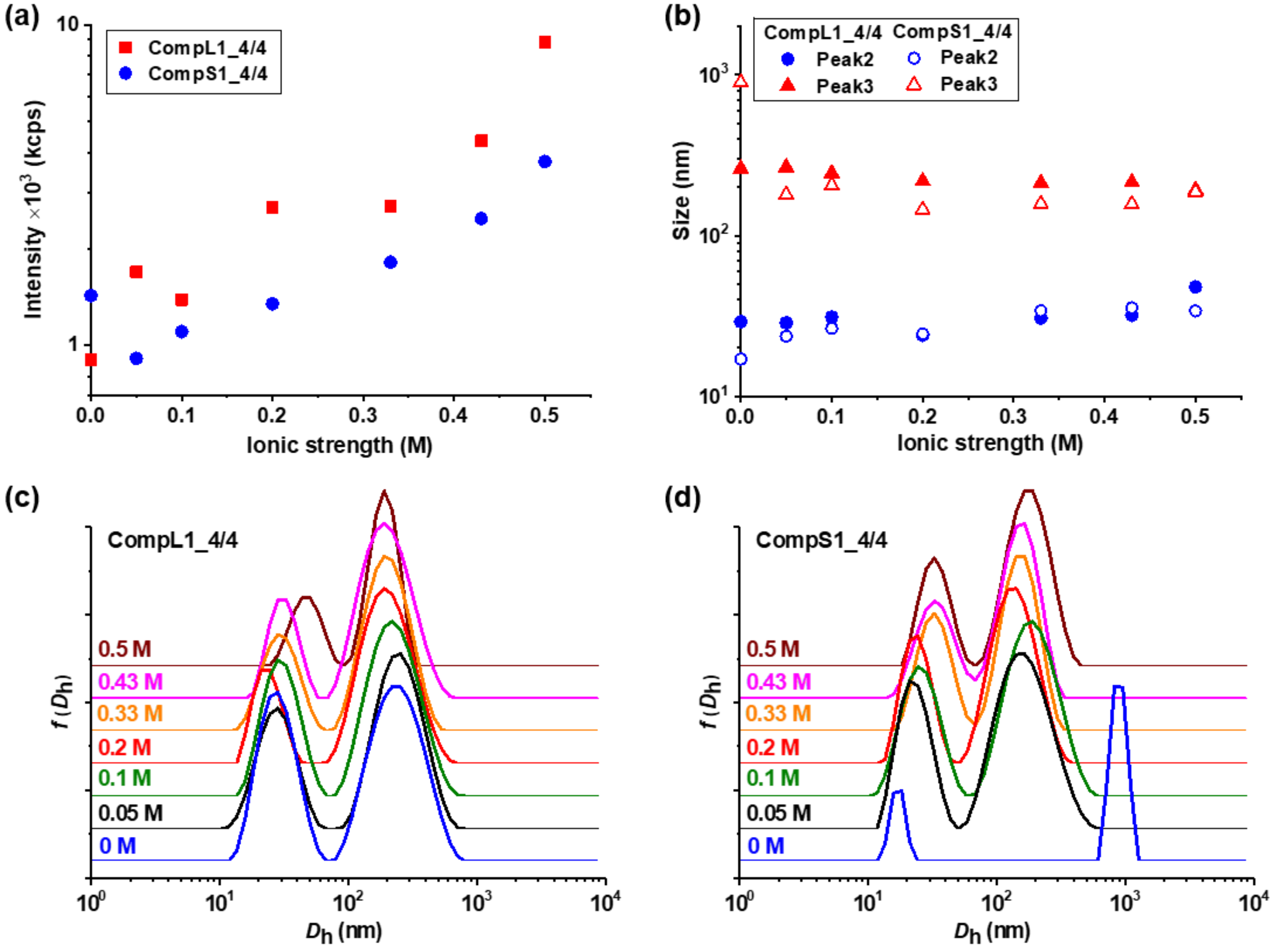

Figure S15. Ionic strength effect on the (**a**) scattered intensity, and (**b**) peaks' size, derived from the corresponding (**c**, **d**) size distributions, for the CompLorS1_4/4 samples.

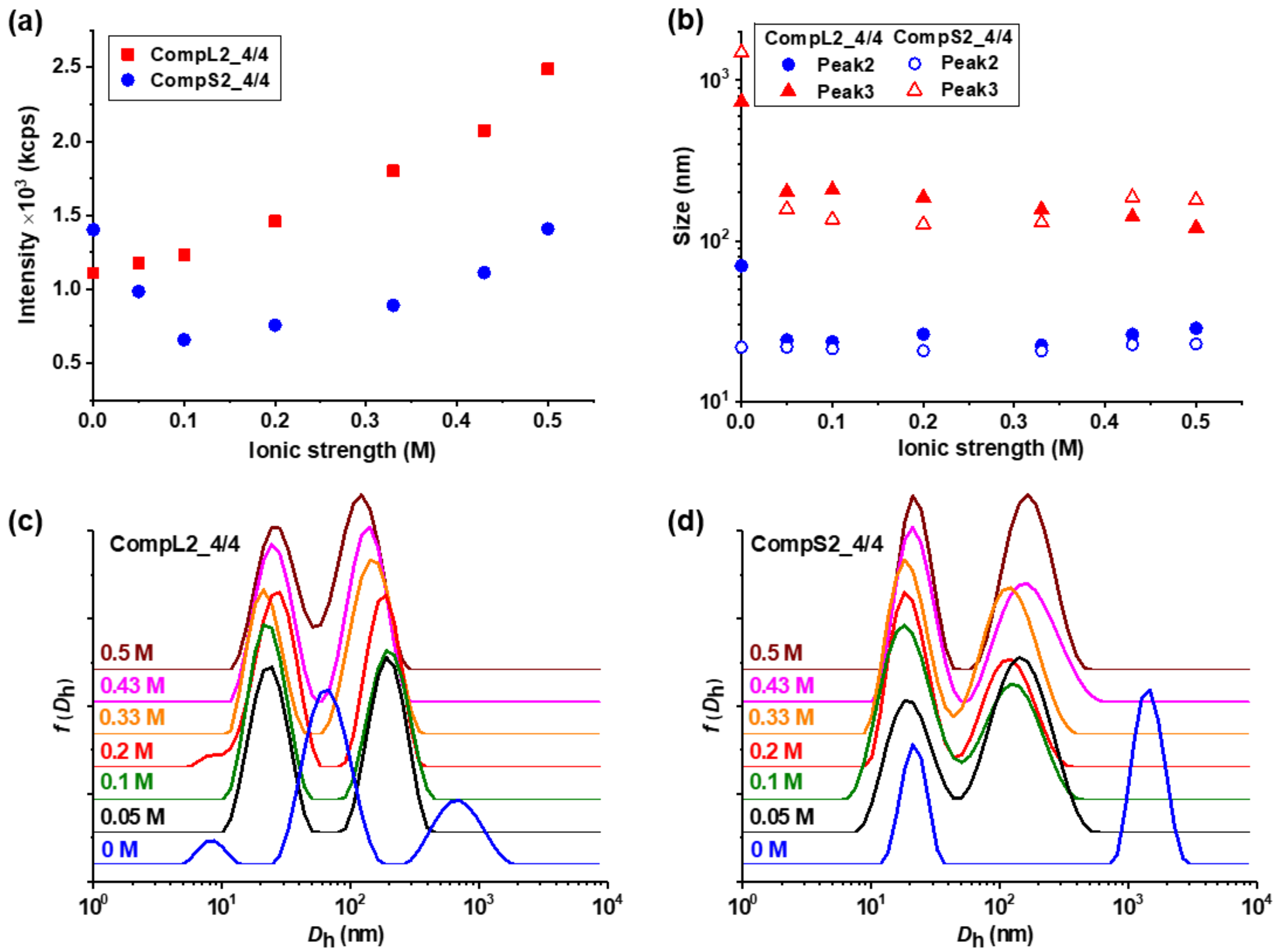

Figure S16. Ionic strength effect on the (**a**) scattered intensity, and (**b**) peaks' size, derived from the corresponding (**c**, **d**) size distributions, for the CompLorS2_4/4 samples.

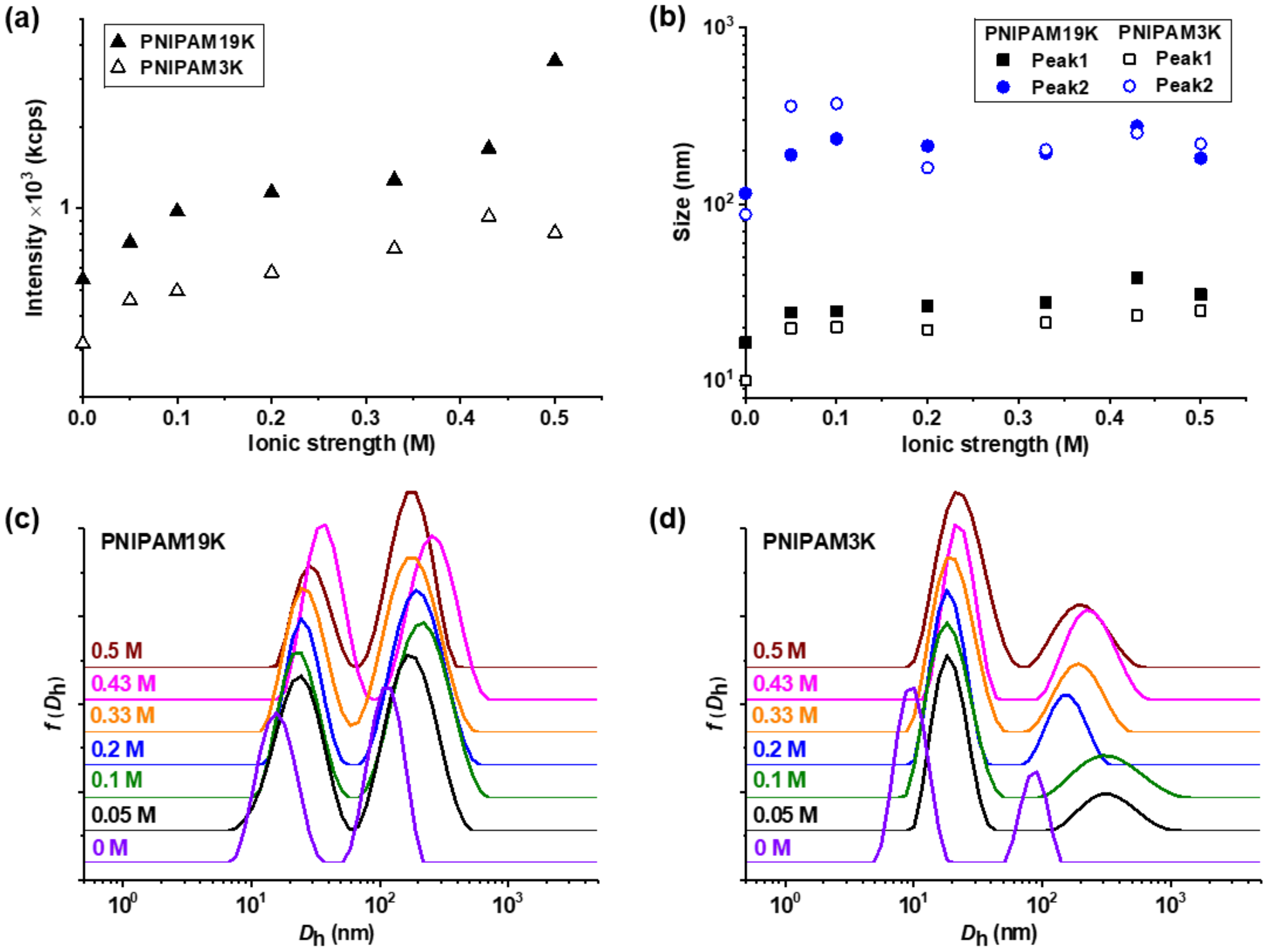

Figure S17. Ionic strength effect on the (**a**) scattered intensity, and (**b**) peaks' size, derived from the corresponding (**c**, **d**) size distributions, for the two PNIPAM samples.

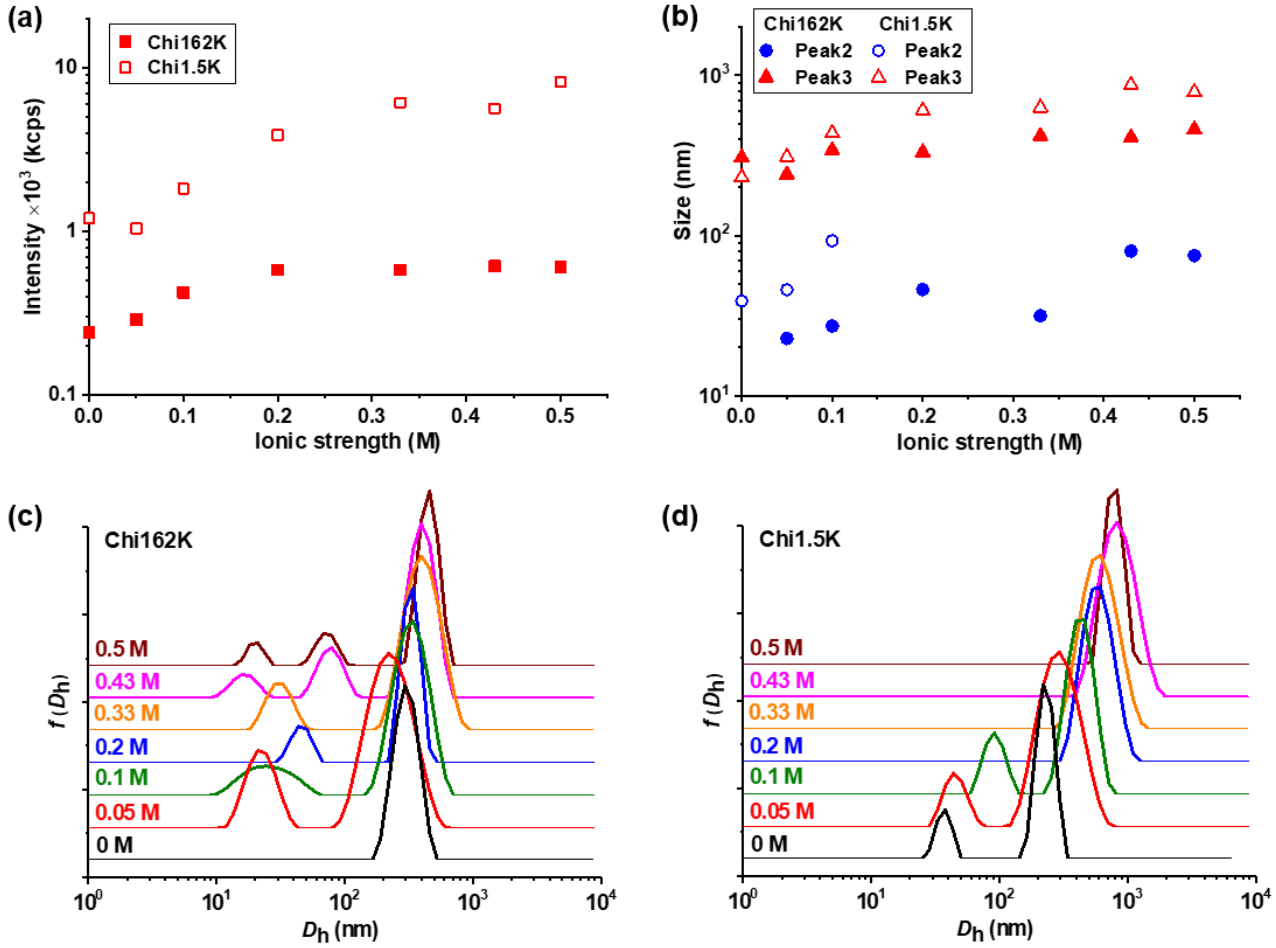

Figure S18. Ionic strength effect on the (**a**) scattered intensity, and (**b**) peaks' size, derived from the corresponding (**c**, **d**) size distributions, for the two chitosan samples.
